# Development and validation of a bean knowledge, attitudes, and behaviors survey in a college population

**DOI:** 10.7717/peerj.21881

**Published:** 2026-10-26

**Authors:** Mohammad Rahanur Alam, Sarah Colby, Iyanuoluwa Oyedeji Oyetunji, Candace Sapp, Jennifer A. Morrow

**Affiliations:** 1 Department of Nutrition and Public Health Sciences, University of Tennessee - Knoxville, Knoxville, TN, United States of America; 2 Department of Educational Leadership & Policy Studies, University of Tennessee - Knoxville, Knoxville, TN, United States of America

**Keywords:** Young adult, Beans, Health knowledge, Attitudes, Practice, Health behavior, Psychometrics, Validity

## Abstract

**Background:**

Despite recommendations for increased bean intake, high nutritional value as a low-cost source of protein, fiber, and other nutrients, and culinary versatility, very few college students regularly consume beans. This study developed and validated a survey to assess knowledge, attitudes, and behaviors related to bean consumption among college students.

**Methods:**

Twenty-six items were generated from a literature review and health behavior theories. An expert panel (*n* = 6) reduced the pool to 20 items assessing students’ bean knowledge, attitudes, and behaviors (content validation). Cognitive interviews (*n* = 7) with college students tested face validity. In all, 982 undergraduate students aged 18–24 were recruited *via* convenience sampling in fall 2023 through an introductory nutrition course. Exploratory factor analysis (EFA) was conducted using principal axis factoring and varimax rotation on a random subsample (*n* = 250). The factor structure was examined using confirmatory factor analysis (CFA) with maximum likelihood estimation (*n* = 732). Missing data was handled *via* Multivariate Imputation by Chained Equations. Internal consistency was assessed using Cronbach’s alpha and McDonald’s omega for multi-item subscales and construct level composite reliability. Cross-validation analysis using 70/30 split, network analysis, and group differences analyses were conducted. Content validity index (CVI) and discriminant validity were also evaluated.

**Results:**

Respondents were primarily female (65.6%), White (90.5%), aged 18–19 (69.6%), and nearly half (46.4%) were freshmen. EFA supported a 4-factor solution (Bean Consumption Behavior, Attitudes and Behavioral Patterns, Perceived Benefits, and Comparative Health Perceptions) explaining 56% variance (Kaiser-Meyer-Olkin (KMO) = .73). CFA showed poor initial model fit (Comparative fit index (CFI) = .840, Tucker-Lewis index (TLI) = .795, root mean square error of approximation (RMSEA) = .096, standardized root mean square residual (SRMR) = .059); after modifications, fit was acceptable (CFI = .914, TLI = .886, RMSEA = .071, SRMR = .047), a significant improvement (Δ*χ*^2^[2] = 239.07, *p* < .001). Cronbach’s alpha for multi-item subscales ranged from .63 to .79 (total-scale alpha = .82; omega = .87). The hypothesized correlated four-factor model fit substantially better than one-factor (CFI = .629) and orthogonal (CFI = .706) alternatives. Factor loadings ranged from .338 to .844; composite reliability from .432 to .660. Cross-validation confirmed model stability across subsamples.

**Conclusions:**

This research produced a validated tool to assess college students’ knowledge, attitudes, and behaviors regarding bean consumption. Results can inform targeted interventions to promote bean intake, an important priority given increasing food insecurity and poor dietary quality among college students.

## Introduction

Poor diet quality among college students is negatively associated with lower academic performance and represents a significant public health concern with serious implications for long-term health and increased risk of developing several chronic diseases (Budnick, Peters & Dowthwaite, 2025; Burrows et al., 2017; Solomou et al., 2023). The transition to university life frequently coincides with the adoption of suboptimal dietary patterns characterized by excessive consumption of processed foods, and limited consumption of nutrient-dense foods, including fruits and vegetables (Wengreen & Moncur, 2009). This dietary transition is also hindered by increasing prevalence of food and nutrition insecurity among college students, with some schools reporting rates higher than 50% of students in the United States experiencing limited or uncertain access to adequate food (Loofbourrow & Scherr, 2023), a condition strongly associated with compromised academic outcomes and adverse health effects (El Zein et al., 2019).

Financial constraints during college commonly result from escalating tuition costs, housing expenses, and food prices, all of which are primary contributors to food and nutrition insecurity among college students (Bruening et al., 2017). Despite the implementation of numerous interventions, programs, and policies designed to address food and nutrition security within this population, sustainable solutions remain elusive (Loofbourrow & Scherr, 2023). One potentially efficacious yet underutilized strategy involves promoting legumes (referred to as beans in this article) as cost-effective, nutrient-dense foods that can simultaneously address nutritional requirements and budgetary limitations.

Beans represent an optimal food category for addressing these multifaceted challenges (Mitchell et al., 2021). Beans provide exceptional nutritional value, including high-quality plant protein, dietary fiber, essential micronutrients, and bioactive compounds, while remaining an economically accessible alternative protein source to higher priced meat (Mudryj, Yu & Aukema, 2014). Substantial evidence suggests that increased legume consumption may be an effective dietary strategy for preventing obesity and reducing the risk of chronic diseases, including cardiovascular disease, type 2 diabetes, and certain cancers (Didinger & Thompson, 2022). Despite this compelling evidence, bean consumption remains suboptimal across the general population and is particularly low among college students (Rana et al., 2021; Semba et al., 2021)

The Dietary Guidelines for Americans recommend eating at least 1.5 cups of legumes per week (US Department of Agriculture & US Department of Health and Human Services , 2020); however, fewer than one-third of Americans consume beans on any given day, with the most recent comprehensive analysis showing that less than 20% of the population are at or above the recommendations for beans (Papanikolaou, Slavin & Fulgoni, 2024). and college students have even poorer consumption patterns. Multiple factors contribute to low intake levels, including limited familiarity with bean preparation, insufficient knowledge regarding nutritional benefits, and low cooking self-efficacy (Winham & Hutchins, 2011). Additional barriers include sensory preferences, potential gastrointestinal effects, temporal constraints, and sociocultural factors influencing food choice behaviors (Chambers et al., 2007).

Nutrition education and behavioral interventions have demonstrated effectiveness in improving college students’ diet quality by enhancing food preparation behaviors and fostering positive food-related attitudes (Asher et al., 2022; Hafiz et al., 2023). Although interventions specifically designed to increase bean consumption are warranted, there is a lack of validated assessment tools available that can accurately measure the multifaceted factors that influence bean intake among this population.

The Behavior, Environment, Attitudes, and Nutrition knowledge (B.E.A.N.) survey, developed by Sowers et al. (2018), provides a foundation for assessment; however, the tool was not designed to measure perceived barriers to eating beans and bean-specific cooking skills both of which constitute critical behavioral determinants (Sowers et al., 2018). Additionally, this instrument was designed based on constructs from the Social Cognitive Theory (Bandura, 1986) and does not include important constructs from complementary behavioral theories. Other available surveys focusing on knowledge, attitudes, and behaviors related to beans have not been specifically validated for college student populations (Melendrez-Ruiz et al., 2023; Winham et al., 2020). These limitations underscore the need for a comprehensive, theoretically grounded instrument that captures the complex interplay of factors influencing bean consumption behaviors among college students.

From a public health perspective, beans are a nutrient-dense, low-cost, and environmentally sustainable protein source, and shifting dietary patterns toward greater legume intake is increasingly recognized as a strategy to improve diet quality and reduce reliance on animal protein among young adults. Despite this, no validated instrument exists to measure the determinants of bean consumption in college students, limiting the ability to design and evaluate targeted interventions. The aim of this study was therefore to develop and validate a theoretically grounded survey assessing bean-related knowledge, attitudes, and behaviors among college students.

This manuscript presents the development and validation of a comprehensive survey instrument that assesses knowledge, attitudes, and behaviors related to bean consumption among college students. Constructs from multiple health behavior theories, including the Knowledge-Attitudes-Behavior (KAB) framework (Saunders et al., 2014), the Health Belief Model (Rosenstock, 1974), the Theory of Planned Behavior (Ajzen, 1991), the Social Cognitive Theory (Bandura, 1986), and the Social Comparison Theory (Festinger, 1954), were used in the development of this instrument to provide a nuanced understanding of factors that facilitate or impede bean consumption in the college student population. The development of such a validated tool provides researchers with the means to evaluate the effectiveness of theory-driven interventions seeking to promote bean consumption as a strategy to improve diet quality and/or address food insecurity among college students.

## Materials & Methods

### Study design and participants

This cross-sectional study aimed to develop and validate a survey instrument assessing college students’ knowledge, attitudes, and behaviors related to bean consumption. Undergraduate students aged 18–24 were recruited from one large US Southeastern University using convenience sampling. Recruitment was conducted through a general undergraduate nutrition class in the beginning of the fall 2023 semester. The target sample size was determined based on guidelines recommending 5–10 participants per item for factor analysis (Comrey & Lee, 1992). Given an initial pool of 26 items and complexity of scale, a recruitment goal of 200–250 participants was set for Exploratory Factor Analysis (EFA) and 650–750 participants for Confirmatory Factor Analysis (CFA). In addition to the newly developed bean-related survey items, food insecurity was assessed using the United States Department of Agriculture (USDAs) six-item Food Security Survey Module (Blumberg et al., 1999), which classified participants as food secure or food insecure. Food insecurity, defined as the limited or uncertain availability of nutritionally adequate food or the inability to acquire food in socially acceptable ways (Blumberg et al., 1999), was included given its established relationship with food consumption behavior among college students (Bruening et al., 2017). The short Healthy Eating Index (sHEI) survey tool was also administered to assess overall diet quality (Colby et al., 2020).

### Survey development

The initial item pool was generated through a structured narrative review of the literature on bean- and legume-related dietary knowledge, attitudes, and behaviors and on existing dietary behavior instruments. PubMed and Scopus were searched from database inception through January 2023 using combinations of the terms (“bean*” OR “legume*” OR “pulse*”) AND (“knowledge” OR “attitude*” OR “behavior*” OR “consumption” OR “intake”) AND (“survey” OR “questionnaire” OR “scale” OR “instrument” OR “validation”). Reference lists of relevant articles were hand-screened to identify additional instruments. Records were retained if they described a survey, scale, or questionnaire assessing legume- or bean-related knowledge, attitudes, practices, or consumption, or measured determinants of legume intake in adult or college-aged populations; records that were not in English or were unrelated to dietary assessment were not considered. Because the objective was item generation rather than quantitative synthesis, a structured narrative rather than systematic review approach was used, and no formal risk-of-bias appraisal or data pooling was performed. Candidate items and constructs were extracted from the identified instruments—including the B.E.A.N. survey (Sowers et al., 2018) and other legume- and plant-based dietary measures (Melendrez-Ruiz et al., 2023; Winham et al., 2020)—and mapped to five constructs and their underlying health behavior theories (Fig. 1), yielding an initial pool of 26 items. Bean-related knowledge was assessed using questions about types of beans, serving sizes, health benefits, nutritional awareness, and preparation methods. Bean consumption behavior was examined through questions about frequency and contextual factors, including consumption as snacks, side dishes, or main dishes. Attitudes were evaluated using questions on preferences, perceived barriers (including taste and cooking time), perceived benefits (including health, financial, and environmental advantages associated with bean consumption) and comparative health perceptions (participants’ views on the healthiness of beans relative to alternative protein sources to meat).

**Figure 1:**
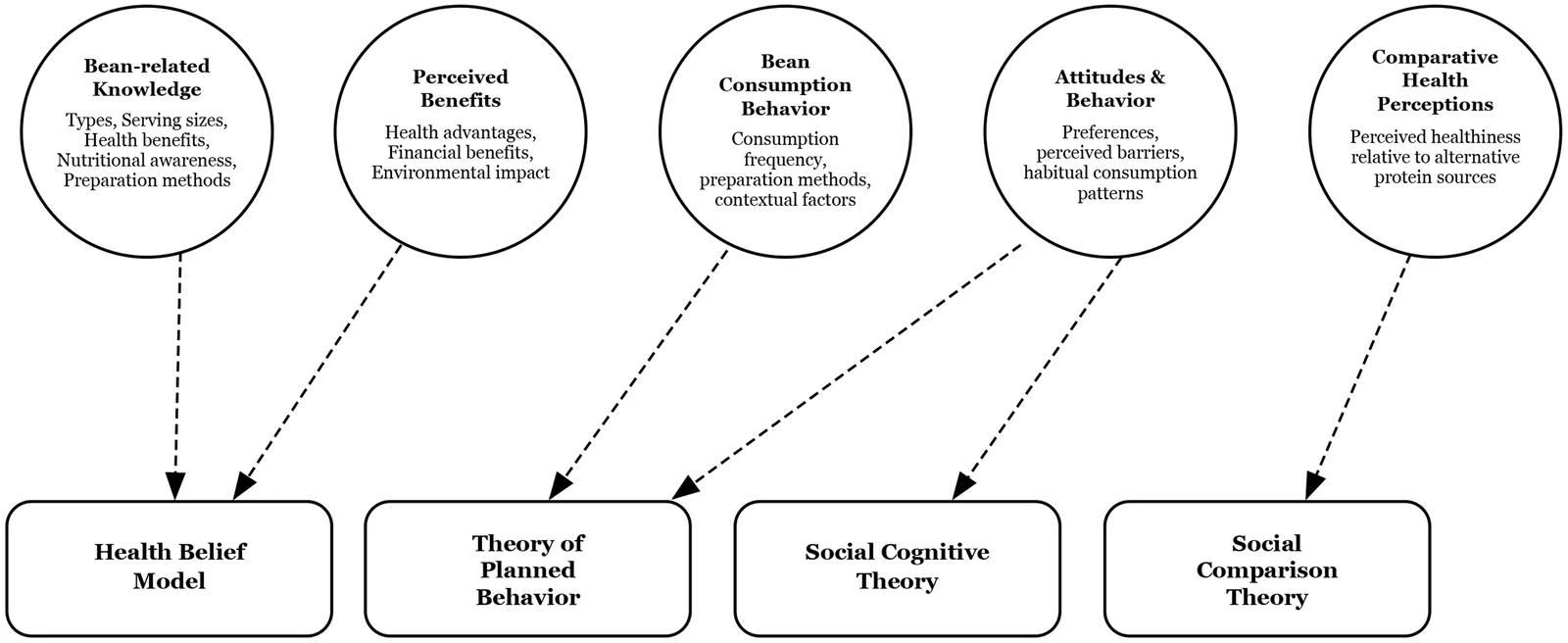
Theoretical framework linking survey constructs to health behavior theories. Theoretical framework mapping five survey constructs (circles) to their corresponding behavioral theories (rectangles). Bean-related Knowledge and Perceived Benefits informed the Health Belief Model; Bean Consumption Behavior informed the Theory of Planned Behavior; Attitudes & Behavior informed both the Theory of Planned Behavior and Social Cognitive Theory; and Comparative Health Perceptions informed Social Comparison Theory. Each construct’s constituent subdomains are listed within its respective circle, illustrating how the instrument’s content was grounded in established health behavior theory.

These five constructs were theoretically grounded in established health behavior frameworks (Fig. 1). The Health Belief Model (Rosenstock, 1974) informed bean-related knowledge and perceived benefits items, in consideration of how awareness motivates behavior change. The Theory of Planned Behavior (Ajzen, 1991) guided bean consumption behavior and attitudes measures, considering how attitudes, subjective norms, and perceived control predict intentions. Social Cognitive Theory (Bandura, 1986) influenced attitudes and behavioral pattern items, highlighting self-efficacy and outcome expectations in sustaining consumption behaviors. Social Comparison Theory (Festinger, 1954) informed comparative health perception items, exploring how relative food comparisons shape dietary choices.

This framework demonstrates the theoretical grounding of survey constructs in established health behavior theories. Each construct was developed based on validated theoretical principles, increasing likelihood that constructs measured would be factors influencing bean consumption behavior. The integration of multiple theoretical perspectives provided a robust foundation for understanding complex dietary behaviors.

### Content validation

A panel of six experts was purposively assembled to evaluate content validity. All panel members held a doctoral degree (PhD), and their expertise spanned nutrition and dietetics, public health, and survey and measurement methodology. Panelists were drawn from multiple US universities and were selected based on relevant subject-matter knowledge and prior experience with dietary behavior assessment and instrument development. Reviewers evaluated the initial 26 items independently, without conferring with one another or with the research team during the rating process. Experts rated each item on a 4-point scale (1 = not relevant, 4 = highly relevant). The content validity index (CVI) was calculated for each item and the overall scale (Polit & Beck, 2006). Items with a CVI < 0.78 were either revised or removed (Lynn, 1986), resulting in a refined 24-item survey. The survey development and validation process is shown in Fig. 2.

**Figure 2:**
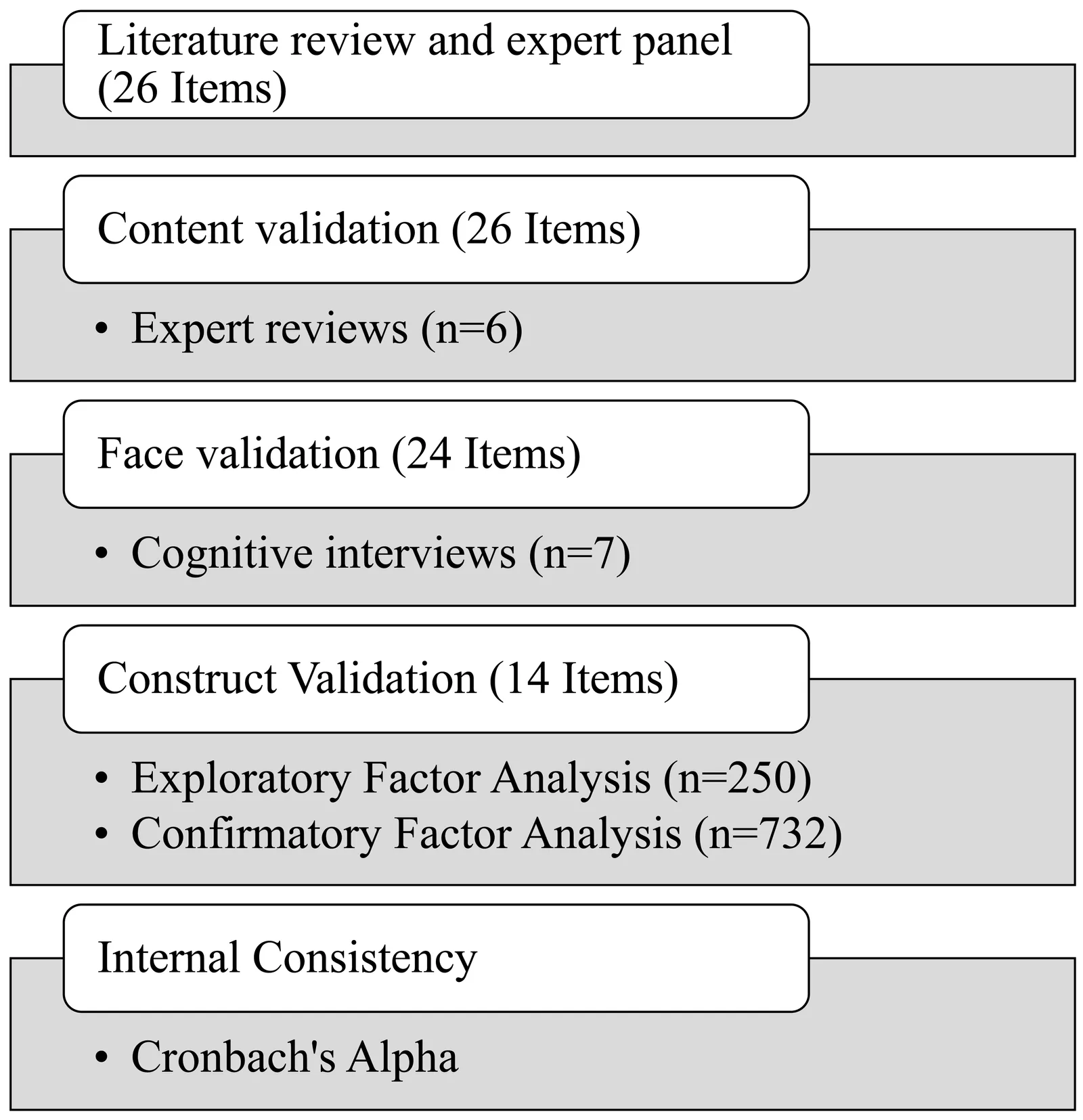
Survey development and validation process. Instrument development and validation process across five sequential phases, beginning with a literature review and expert panel that generated 26 candidate items. Items were progressively refined through content validation *via* expert reviews (*n* = 6, retaining 26 items) and face validation *via* cognitive interviews (*n* = 7, reducing to 24 items). Construct validity was then established through exploratory factor analysis (*n* = 250) and confirmatory factor analysis (*n* = 732), yielding a 14-item scale. Internal consistency of the final instrument was evaluated using Cronbach’s Alpha.

### Face validation

Cognitive interviews were conducted with seven undergraduate college students (4 female, 3 male) individually. Participants completed the 24-item survey using a think-aloud protocol, verbalizing their thought processes for each item (Wolcott & Lobczowski, 2021). Minor adjustments to wording were made based on this feedback to improve comprehension.

### Construct validation

Construct validity was assessed in two sequential, complementary stages using independent subsamples to avoid capitalizing on chance. Exploratory factor analysis (EFA) was conducted first to identify the underlying factor structure empirically, without imposing a priori model; confirmatory factor analysis (CFA) was then conducted on a separate subsample to test how well that hypothesized structure fit new data.

### Exploratory Factor Analysis

EFA was performed utilizing a random sample of 250 students to explore the underlying factor structure. Principal axis factoring with varimax rotation was used to maximize interpretability and achieve orthogonal factors (Kaiser, 1958). The number of factors to retain was determined using parallel analysis and the scree plot method (Horn, 1965). Items were removed iteratively: those with primary loadings below 0.40 and those with cross-loadings of 0.32 or higher on multiple factors were excluded sequentially, with the analysis re-run after each removal until a clean structure emerged (Howard, 2016). Specifically, three knowledge items (K1, K2, K3) were removed first due to low or non-significant loadings (*n* = 3 items), followed by items with low communalities or failing to load cleanly (B15, B14, B9; *n* = 3 items), then A8 (*n* = 1 item) for insufficient loading magnitude, and finally B1 and A6 (*n* = 2 items) for not meeting the loading threshold on any factor, with the model reduced from five to four factors at this stage. A10 was removed last (*n* = 1 item) for near-zero communality. A complete list of original items, including those removed during EFA, is provided in Table S1.

### Confirmatory Factor Analysis

CFA was conducted on the remaining sample of 732 students to validate the factor structure identified in the EFA. Maximum Likelihood Estimation (MLE) was employed, and missing data were addressed through multiple imputation using the Multivariate Imputation by Chained Equations (MICE) algorithm (Van Buuren & Groothuis-Oudshoorn, 2011). Model fit was assessed using the following indices and thresholds: Comparative Fit Index (CFI) and Tucker-Lewis Index (TLI) > 0.90, Root Mean Square Error of Approximation (RMSEA) < 0.08, and Standardized Root Mean Square Residual (SRMR) < 0.06 (Hu & Bentler, 1999).

### Internal consistency

Internal consistency for each construct was evaluated using Cronbach’s Alpha, with an alpha value of ≥0.70 indicating acceptable reliability (Nunnally & Bernstein, 1994). Internal consistency was quantified for each multi-item factor using both Cronbach’s alpha and McDonald’s omega; for the two-item factor, the Spearman-Brown coefficient was reported because alpha and omega are not appropriate for two indicators. Construct-level reliability and convergent validity were further evaluated using composite reliability (CR) and average variance extracted (AVE). Discriminant validity among the latent factors was assessed using the Fornell-Larcker criterion, which compares the square root of each factor’s AVE with its inter-factor correlations, and the heterotrait-monotrait (HTMT) ratio of correlations, with values below .85 indicating adequate discriminant validity. To confirm that the four-factor structure was the best-fitting representation of the data, the hypothesized correlated four-factor model was compared with a unidimensional (one-factor) model and an orthogonal four-factor model using the Akaike information criterion (AIC), Bayesian information criterion (BIC), comparative fit indices, and the scaled chi-square difference test.

### Statistical analysis

All statistical analyses were conducted using R version 4.4.1. Descriptive statistics were generated to summarize participant demographics. EFA was performed using the ‘psych’ package (Revelle, 2017), while CFA was conducted using the ‘lavaan’ package (Rosseel, 2012). Multiple imputation for missing data was implemented using the ‘mice’ package (Van Buuren & Groothuis-Oudshoorn, 2011). Reliability coefficients (Cronbach’s alpha and McDonald’s omega), composite reliability, average variance extracted, and HTMT ratios were computed using the ‘psych’ and ‘semTools’ packages.

### Ethical considerations

The study protocol was approved by the Institutional Review Board (IRB) at the University of Tennessee Knoxville (UTK IRB-23-07396-XP). Participants provided informed consent electronically by reading an online information sheet and indicating their agreement to participate by clicking an ‘I agree’ button before accessing the survey. Responses were anonymized to maintain confidentiality, and data were stored on secure, password-protected servers. Participants were compensated with extra course credit for completing the survey; however, participants did not have to allow their data to be used in research to receive the extra credit.

## Results

Participants were primarily female (65.6%), White (90.5%), between 18–19 years old (69.6%), and first-year students and sophomores comprised over 80% of the study population (Table 1). Food insecurity was prevalent (56.1%), with 35.7% experiencing low (35.7%) and 20.4% experiencing very low (20.4%) food security; 43.9% of participants maintained high or marginal food security.

**Table 1:** Demographic characteristics of the study sample (*N* = 982).

Variable	Category	N	Percent
Gender	Female	644	65.6%
	Male	332	33.8%
	Other/Prefer not to answer	6	0.6%
Age	18–19	683	69.6%
	20–25	299	30.4%
Ethnicity	American Indian or Alaska Native	5	0.5%
	Asian	25	2.5%
	Black or African American	32	3.3%
	Native Hawaiian or Pacific Islander	1	0.1%
	White	889	90.5%
	Hispanic or Latino	14	1.4%
	Mixed race	13	1.3%
	Prefer not to answer	3	0.3%
Year in college	Freshman	456	46.4%
	Sophomore	350	35.6%
	Junior	118	12.0%
	Senior	58	5.9%
Food security status	High/marginal food security	431	43.9%
	Low Food Security	351	35.7%
	Very Low Food Security	200	20.4%

**Notes.**

Demographics reflect the combined sample used for validation analyses. Percentages may not sum to 100% due to missing information.

An exploratory factor analysis (EFA) was performed with 250 participants to investigate the underlying structure of bean consumption behaviors and attitudes. Prior to analysis, variables with more than 50% missing data were excluded (*n* = 2 variables), cases with missing values were removed *via* listwise deletion (*n* = 18 cases), and items with zero variance were omitted (*n* = 1 item). The dataset was deemed suitable for factor analysis, as indicated by a significant Bartlett’s test of sphericity, *χ*^2^(276) = 1,079.37, *p* < .001, and a Kaiser-Meyer-Olkin (KMO) measure of sampling adequacy of .73, which exceeds the recommended threshold of .60.

Examination of the scree plot (Fig. S1) and results of the parallel analysis initially suggested the retention of five factors. However, after iterative model refinement—including sequential removal of items with primary loadings below 0.40 or cross-loadings ≥0.32, re-running the analysis after each removal, and consideration of statistical fit indices and theoretical interpretability—a four-factor solution was selected as the most parsimonious and interpretable structure. Ten items were removed across five iterative rounds (see Table S1 for the full item list). The final model included 14 items across four factors containing 4, 5, 3, and 2 items, respectively, and accounted for 56% of the total variance.

The four identified factors were: (1) Consumption (4 items; factor loadings range: .58 to .79), reflecting sensory attitudes toward beans and frequency of basic consumption; (2) Purchasing (5 items; factor loadings range: .55 to .75), encompassing various contexts and acquisition behaviors, such as consuming beans in different forms and purchasing canned beans; (3) Perceived Benefits (3 items; factor loadings range: .46 to .89), measuring perceptions of health and financial advantages associated with bean consumption; and (4) Comparative Evaluations (2 items; factor loadings range: .52 to .98), representing comparative assessments of beans *versus* meat with regard to environmental sustainability and healthfulness (Table 2).

**Table 2:** Final factor loadings and communalities. Exploratory factor analysis pattern matrix for the 14-item survey instrument (*n* = 250), yielding four factors: Consumption (Factor 1), Purchasing (Factor 2), Perceived benefits (Factor 3), and Comparative evaluations (Factor 4). Loadings represent item-factor associations after orthogonal rotation; *h*^2^ = communality (variance explained by all factors); *u*^2^ = uniqueness (unexplained variance); com = item complexity. Factor 4 demonstrated the strongest item loadings (A4, *λ* = 0.98), while A5 showed relatively low communality (*h*^2^ = 0.375), suggesting weaker alignment with its assigned factor.

Item	ML3	ML4	ML2	ML1	h2	u2	com
**Factor 1: Consumption**							
A9: How much do you agree with the following statement? - I do not like the texture of beans.	0.79				0.68	0.32	1.2
A3: How much do you agree with the following statement? - I like the taste of beans.	−0.70				0.711	0.289	1.9
B6: How often do you consume beans as a side dish?	0.59				0.451	0.549	1.6
B2: On average, how often do you eat beans/products made from beans?	0.58				0.41	0.59	1.4
**Factor 2: Purchasing**							
B5: How often do you consume beans as a dessert?		0.75			0.565	0.435	1
B4: How often do you buy canned beans?		0.66			0.539	0.461	1.5
B7: How often do you consume beans as a main-dish?		0.59			0.386	0.614	1.2
B8: How often do you use beans as an ingredient during cooking?		0.57			0.446	0.554	1.7
B3: How often do you consume beans as a dip?		0.55			0.428	0.572	1.8
**Factor 3: Perceived Benefits**							
A2: How much do you agree with the following statement? - Beans are affordable.			0.89		0.816	0.184	1
A1: How much do you agree with the following statement? - Beans are good for my health.			0.87		0.777	0.223	1
A5: How much do you agree with the following statement? - Buying beans saves me money.			0.46		0.375	0.625	2.5
**Factor 4: Comparative Evaluations**							
A4: How much do you agree with the following statement? - Beans are better for the environment than meat.				0.98	0.995	0.005	1.1
A7: How much do you agree with the following statement? - Beans can be as healthy as meat.				0.52	0.287	0.713	1.2

**Notes.**

h2 communality u2 uniqueness com complexity

The four-factor model demonstrated acceptable fit (RMSR = .04; df-corrected RMSR = .06). Factor score adequacy was evidenced by strong correlations between regression scores and factors (*r* = .87 to 1.00) (see Table S2 for detailed EFA fit statistics).

Based on the EFA results, a four-factor confirmatory model was specified with 14 observed indicators loading on their respective latent factors as identified in the exploratory phase. The initial model demonstrated poor fit to the data (*χ*^2^[71] = 546.77, *p* < .001; CFI = .840; TLI = .795; RMSEA = .096 90% CI [.088–.103]; SRMR = .059), indicating the need for model modification. Examination of modification indices revealed theoretically justified areas for model improvement. The largest modification indices suggested adding residual covariances between conceptually related items: A9 (texture dislike) and A3 (taste preference), both measuring sensory attitudes toward beans, and A2 (affordability) and A1 (health benefits), both reflecting positive bean attributes.

The modified model incorporating these residual covariances showed substantially improved fit (*χ*^2^[69] = 325.19, *p* <  .001; CFI = .914; TLI = .886; RMSEA = .071 90% CI [.064–.079]; SRMR = .047) (Table 3). The chi-square difference test confirmed significant improvement (Δ*χ*^2^[2] = 239.07, *p* < .001).

**Table 3:** Model fit indices for CFA models. The Modified Model demonstrated the best overall fit, meeting acceptable thresholds for CFI (0.914), TLI (0.886), RMSEA (0.071), and SRMR (0.047) following the addition of two residual covariances identified in the Initial Model. The Orthogonal Model, which constrained all interfactor correlations to zero, fit the data substantially worse across all indices, supporting the retention of correlated factors in the final solution.

**Model**	*χ* ^2^	**df**	*P*	**CFI**	**TLI**	**RMSEA [90% CI]**	**SRMR**
Initial Model	546.77	71	<.001	0.84	0.795	0.096 [0.088, 0.103]	0.059
Modified Model	325.19	69	<.001	0.914	0.886	0.071 [0.064, 0.079]	0.047
Orthogonal Model	949.81	75	<.001	0.706	0.643	0.126 [0.119, 0.133]	0.177

**Notes.**

*χ*^2^ chi-square df degrees of freedom CFI comparative fit index TLI Tucker-Lewis index RMSEA root mean square error of approximation CI confidence interval SRMR standardized root mean square residual

The path diagram (Fig. 3) illustrates the four-factor model consisting of Consumption, Purchasing, Perceived Benefits, and Comparative Evaluations as latent factors. All standardized factor loadings were statistically significant (*p* < .001) and ranged from .338 (B5) to .844 (A4). The Consumption factor showed loadings ranging from .338 (B5) to .635 (B4); while most indicators were adequate, B5 fell below the .40 threshold recommended by Hair et al. (2019). The Purchasing factor included loadings from .488 (A9) to .759 (B6), with A3 exhibiting a negative loading (−0.695). The Perceived Benefits factor demonstrated moderate loadings (.556 to .753), with A5 contributing most strongly. The Comparative Evaluations factor was anchored by A4 (.844), with A7 loading at .483. Despite some indicators falling below conventional cutoffs, all items were retained to preserve content coverage and theoretical representativeness.

**Figure 3:**
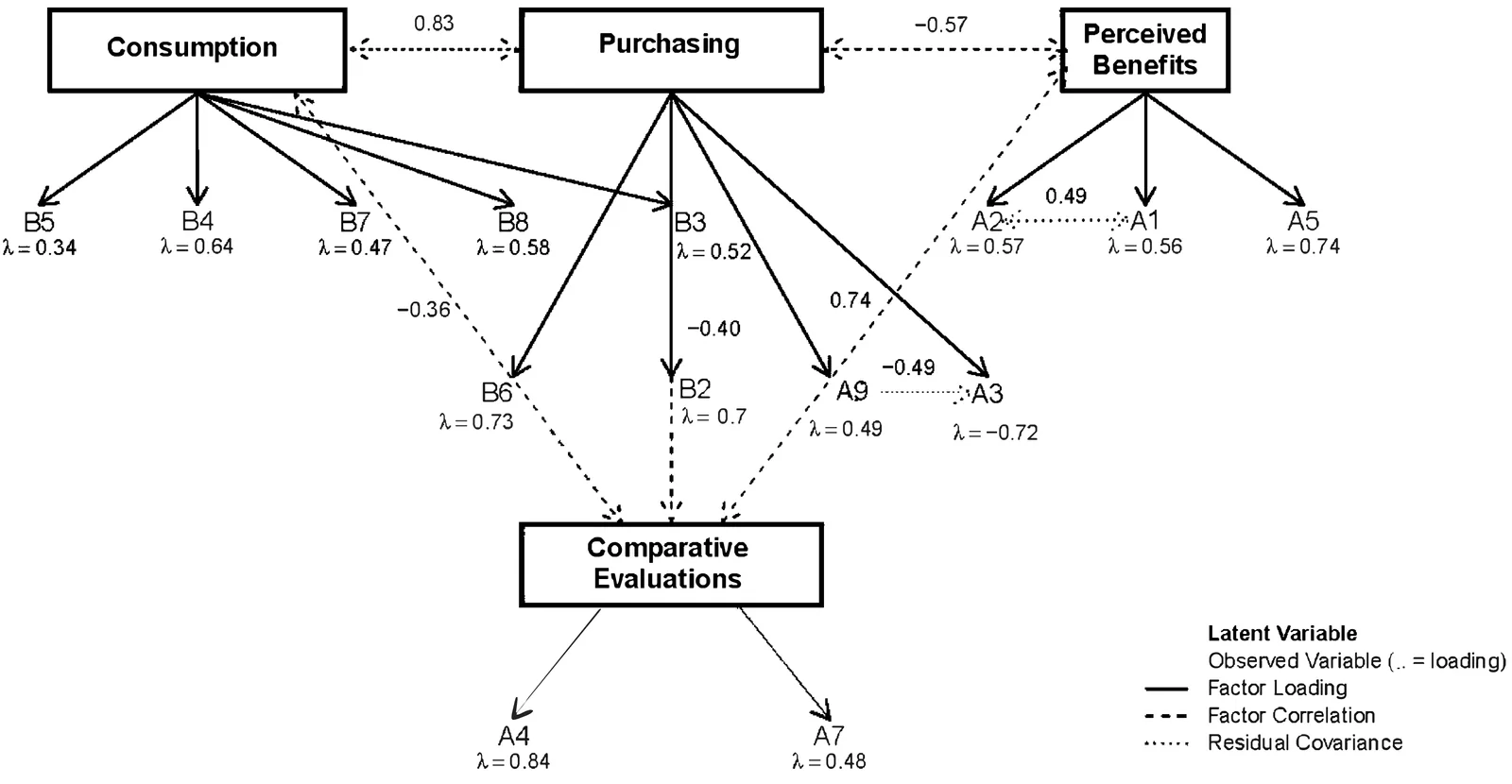
Confirmatory factor analysis path diagram of survey constructs. Confirmatory factor analysis path diagram depicting the structure and inter-relationships of four latent constructs: Bean Consumption Behavior, Attitudes and Behavioral Patterns, Perceived Benefits, and Comparative Health Perceptions. Solid arrows represent factor loadings (*λ* ) from each latent variable to its observed indicators, ranging from weak (*λ* = 0.34, B5) to strong (*λ* = 0.84, A4). Dashed lines indicate factor correlations between latent constructs, notably a strong positive association between Bean Consumption Behavior and Attitudes and Behavioral Patterns (*r* = 0.83) and a moderate negative correlation between Attitudes and Behavioral Patterns and Perceived Benefits (*r* = −0.57). Dotted lines denote residual covariances between select indicator pairs (A2–A1, *r* = 0.49; A9–A3, *r* = −0.49), suggesting shared method variance or conceptual overlap beyond what the latent factors account for. Cross-loadings from Attitudes and Behavioral Patterns onto indicators nominally assigned to other factors (*e.g.*, B6, B2, A9) are shown with dashed arrows and negative path coefficients (−0.36, −0.40, −0.49), reflecting suppression effects within the model.

Eight correlated residuals were specified based on sequential evaluation of modification indices, with each addition assessed for theoretical plausibility. The correlated residual between A1 (health beliefs) and A2 (affordability beliefs) was specified to account for shared method variance, as both items reflect widely held normative beliefs that produced similar endorsement patterns (*M* = 4.10 and 4.17, respectively; SD = 0.68 for both). The remaining correlated residuals were attributable to content adjacency or overlapping item phrasing within their respective factors.

Factor correlations spanned from −0.532 to 0.816, with the strongest positive correlation observed between Bean Consumption Behavior and Attitudes and Behavioral Patterns (*r* = 0.816). The strongest negative correlation was between Attitudes and Behavioral Patterns and Perceived Benefits (*r* = −0.532). Composite reliability (CR) values ranged from 0.432 to 0.660, indicating acceptable internal consistency for most factors, with the exception of Attitudes and Behavioral Patterns. A residual analysis revealed that only 3.3% of residuals exceeded —0.1—, indicating reasonably good local fit. An orthogonal model was also tested for comparison, demonstrating significantly poorer fit than the modified correlated factors model (Δ*χ*^2^[6] = 370.13, *P* < .001). While the modified four-factor model demonstrated improved fit over both the initial and orthogonal models, the suboptimal AVE values necessitate further refinement of the measurement items to enhance construct validity in future research.

Internal consistency of the multi-item subscales ranged from acceptable to good. Cronbach’s alpha and McDonald’s omega were .63 and .65, respectively, for Bean Consumption Behavior; .79 and .80 for Attitudes and Behavioral Patterns; and .73 and .77 for Perceived Benefits. For the two-item Comparative Health Perceptions factor, for which alpha and omega are not appropriate, the Spearman-Brown coefficient was .58. Reliability for the full 14-item scale was high (alpha = .82; omega = .87), indicating that the instrument functions consistently as an overall measure while the lower coefficients for the shorter subscales reflect their small number of items.

To justify the four-factor solution, competing measurement models were compared. The hypothesized correlated four-factor model (AIC = 23,466.75; BIC = 23,696.54; CFI = .914; RMSEA = .071; SRMR = .047) fit the data substantially better than a unidimensional one-factor model (AIC = 24,307.59; BIC = 24,500.61; CFI = .629; RMSEA = .140; SRMR = .095) and an orthogonal four-factor model (AIC = 24,079.38; BIC = 24,281.59; CFI = .706; RMSEA = .126; SRMR = .177) (Table 4). The correlated four-factor model yielded the lowest AIC and BIC, and a scaled chi-square difference test confirmed that it fit significantly better than the one-factor model (Δ*χ*^2^[8] = 555.88, *p* < .001). These comparisons support the retention of four correlated factors over both a single general factor and a fully independent factor structure.

**Table 4:** Standardized factor loadings, reliability, and validity measures for the modified CFA model. Standardized factor loadings, average variance extracted (AVE), and composite reliability (CR) for the four-factor modified CFA model (*n* = 732). All loadings were statistically significant (*p* < .001), though several fell below the conventional 0.70 threshold, most notably B5 (*λ* = 0.338) and A7 (*λ* = 0.483). AVE values ranged from 0.269 to 0.473, falling below the recommended 0.50 cutoff for all factors, indicating modest convergent validity. CR values were acceptable for Bean Consumption Behavior (0.639), Perceived Benefits (0.660), and Comparative Health Perceptions (0.626), but notably low for Attitudes and Behavioral Patterns (0.432), partly attributable to the negatively loaded item A3, reflecting the mixed directional nature of that construct.

**Factor**	**Item**	**Standardized loading**	**AVE**	**CR**
Bean Consumption Behavior	B5	0.338	0.269	0.639
	B4	0.635		
	B7	0.468		
	B8	0.582		
	B3	0.522		
Attitudes and Behavioral Patterns	B6	0.759	0.458	0.432
	B2	0.732		
	A9	0.488		
	A3	−0.695		
Perceived Benefits	A2	0.565	0.398	0.66
	A1	0.556		
	A5	0.753		
Comparative Health Perceptions	A4	0.844	0.473	0.626
	A7	0.483		

**Notes.**

All factor loadings are significant at *P* < .001.

AVE Average Variance Extracted CR Composite Reliability

Network analysis using correlation-based networks revealed strong clustering patterns consistent with the four-factor structure (Fig. S2). Central nodes included items related to bean consumption frequency (B4, B5) and perceived benefits (A1, A2).Cross-validation analysis using a 70/30 split demonstrated excellent model stability and generalizability. Factor scores were computed using a modified mean substitution approach. Item A9 (texture dislike) was reverse scored (1=5, 2=4, 3=3, 4=2, 5=1). Frequency items (B2–B8) were scored directly (1–5), with “Choose not to answer” responses (value 6) recoded to 0 and excluded. All agreement items (A1–A5, A7) were scored directly. The complete item scoring scheme is provided in Table S4.

Factor scores were computed using a modified mean substitution approach that accounts for the diverse item types and missing response patterns (DiStefano, Zhu & Mindrila, 2009). Item A9 was reverse coded (5, 4, 3, 2, 1) so that higher scores reflect more favorable attitudes toward beans. Frequency items (B2–B8) that received a “Choose not to answer” response (coded as 6) were recoded to 0 and excluded from calculations. All remaining agreement items (A1–A5, A7) were scored directly (1–5). For each factor, scores represent the mean of valid (non-zero) responses across constituent items, preserving the original 1–5 scale metric while accommodating partial response patterns. An overall composite score was computed as the unweighted mean of the four factor scores. In the CFA sample (*N* = 732), factor score means were: Consumption = 4.31 (SD = 0.52), Purchasing = 3.04 (SD = 0.49), Perceived Benefits = 3.89 (SD = 0.59), and Comparative Evaluations = 3.17 (SD = 0.77). The complete item-by-item scoring scheme is provided in Table S4.

The scoring system was applied to examine group differences across demographic characteristics, providing evidence of discriminant validity. Significant differences were observed by gender (F[2,979] = 3.87, *p* = .021; *η*^2^ = .008), with females scoring higher (*M* = 39.2, SD = 4.3) compared to males (*M* = 38.1, SD = 4.5). Nonparametric Kruskal–Wallis testing confirmed this pattern (*χ*^2^ = 8.33, *p* = .016). Marginally significant differences were observed by food security status (F[2,979] = 2.65, *p* = .071; *η*^2^ = .005), with students experiencing very low food security showing slightly different scores (*M* = 38.1, SD = 4.6) compared to those with high/marginal security (*M* = 38.8, SD = 4.3), confirmed by Kruskal–Wallis testing (*χ*^2^ = 6.52, *p* = .038). No significant differences were found by age group (F[1,980] = 0.64, *p* = .425) or academic year (F[3,978] = 0.54, *p* = .657).

Correlational analysis with the short Healthy Eating Index revealed minimal association (*r* = .027, *p* = .398), indicating that the bean consumption attitudes and behaviors measured by this instrument represent constructs distinct from general dietary quality as assessed by the sHEI. This finding supports discriminant validity, as the bean-specific survey captures unique variance not accounted for by a broad diet quality measure. The pattern of group differences further supports discriminant validity (Fig. S3).

Discriminant validity among the latent factors was further examined using the Fornell-Larcker criterion and HTMT ratios. All HTMT values were below the .85 threshold (range = .26 to .71), supporting discriminant validity. The Fornell-Larcker criterion was met for most factor pairs but was not satisfied for two strongly related pairs—Bean Consumption Behavior and Attitudes and Behavioral Patterns (*r* = .82) and Perceived Benefits and Comparative Health Perceptions (*r* = .73)—for which the inter-factor correlation exceeded the square root of the average variance extracted. This pattern is consistent with the comparatively low AVE values noted above and indicates that, although the factors are empirically distinguishable by the HTMT criterion, the conceptual overlap between behavior and behavioral attitudes, and between perceived and comparative benefits, warrants further item refinement in future work.

## Discussion

This study successfully developed and validated a survey instrument to assess the knowledge, attitudes, and bean consumption behavior among college students. This study contributes uniquely to the literature grounding its instrument development in the Knowledge-Attitudes-Behavior (KAB) framework (Saunders et al., 2014) and including constructs from four additional health behavior theories:, including the Health Belief Model (Rosenstock, 1974), the Theory of Planned Behavior (Ajzen, 1991), the Social Cognitive Theory (Bandura, 1986), and the Social Comparison Theory (Festinger, 1954). These additions build on the work of Sowers et al. (2018) that drew constructs from only the Social Cognitive Theory. The inclusion of constructs from multiple theories accounts for a higher variance in behavior initiation/sustenance than a single theoretical approach (Kapukotuwa et al., 2024). This, therefore, helps to provide a more nuanced, behaviorally informed tool that supports theory-driven assessment of interventions developed for a college population.

This tool provides a way to not only assess the perceived benefits of beans, but also to assess the comparative environmental and health benefits of consuming beans *versus* meat. Increasing evidence shows that replacing meat with legumes, such as beans, has health and environmental benefits (Kaartinen et al., 2023; Sranacharoenpong et al., 2015), and as such was an important construct to include. This newly developed tool provides an opportunity to both assess participants’ baseline attitudes and knowledge related to the use of beans as a meat replacement but also to determine the effectiveness of an intervention that has that protein replacement as an outcome of interest. Including a construct that prompts respondents to compare the benefits of consuming beans with those of meat represents a methodological innovation in our survey design. Another key innovation of the newly developed tool is the inclusion of a broader range of bean consumption forms compared to the previous tool of Sowers et al. (2018).

The questions allow for participants’ consumption patterns of beans as a side dish or dessert, such as in hummus, which may be another desired outcome in future bean promotion interventions. The strong correlation between Consumption and Purchasing (*r* = .816) indicates that changing attitudes toward beans may directly translate to behavioral modifications. These newly developed questions allow researchers to tailor intervention design to be align with evidence that beans are commonly consumed as part of composite meals or culturally specific dishes rather than in isolation (Mitchell et al., 2009; Winham, Armstrong Florian & Thompson, 2016). Having a broader coverage of bean consumption also enhances inclusivity, capturing a range of culinary practices across ethnic and socioeconomic groups, a critical consideration for equitable dietary assessment and public health planning (Ali, Lin & Yi, 2023). By recognizing these nuances in consumption patterns, the newly developed tool reflects real-world dietary behavior and supports culturally sensitive interventions aimed at increasing bean intake among college students of different backgrounds.

Furthermore, the inclusion of an objective content validity index of 0.78 or higher, from the responses of six expert reviewers, improves the rigor and content validity of the new tool (Polit & Beck, 2006). Convergent validity using average variance extracted (AVE) yielded values ranging from 0.269 to 0.473, which fall below the recommended threshold of 0.5 for all factors (Fornell & Larcker, 1981). This suggests potential issues with the construct’s convergent validity. This is another addition to the literature as the tool of Sowers et al. (2018) did not assess convergent validity.

The factor structure identified in this study provides important insights for intervention design and implementation. The four-factor model reveals distinct pathways through which bean consumption behaviors may be influenced, suggesting that interventions should adopt multi-pronged approaches rather than focusing on single determinants (Michie, van Stralen & West, 2011). The strong correlation between bean Consumption behavior and Purchasing (*r* = .816) indicates that changing attitudes toward beans may directly translate to behavioral modifications, supporting attitude-based intervention strategies consistent with the Theory of Planned Behavior (Ajzen, 1991).

The major limitation of this tool is that it is developed and validated specifically with a college student population. Although the demographic profile of participants in the current study is similar to previous studies (Robbins, Spence & Steeves, 2022; Sowers et al., 2018). This current study utilized a larger sample of participants compared to the Sowers’ study, which provided the present study enough power to appropriately test additional hypotheses. Limitations beyond the demographic homogeneity and specificity of the sample included the cross-sectional design, which precludes assessment of test–retest reliability and temporal stability of the factor structure (DeVellis & Thorpe, 2021; Widaman, Ferrer & Conger, 2010). Additionally, the self-report nature of the instrument introduces potential social desirability bias, particularly for items related to environmental consciousness and health awareness (Hebert et al., 1995).

A further limitation concerns the recruitment source. Participants were drawn from a single introductory nutrition course at one institution, and students who enroll in a nutrition course—even one open to all majors—may have greater baseline interest in or awareness of nutrition than the general undergraduate population. The use of extra course credit as an incentive may have additionally shaped who chose to participate. Together with the predominantly female and White composition of the sample, these factors may bias knowledge and attitude responses and constrain generalizability, reinforcing the need for external validation in students recruited through other channels and at other institutions.

The psychometric performance of the present instrument is broadly comparable to that reported for other dietary knowledge, attitude, and behavior tools, including the bean-focused B.E.A.N. survey (Sowers et al., 2018) and legume- and plant-based diet questionnaires developed in non-college populations (Melendrez-Ruiz et al., 2023; Winham et al., 2020). The total-scale reliability observed here (alpha = .82; omega = .87) is consistent with values typically considered acceptable for newly developed dietary measures, while the lower coefficients for the briefer subscales mirror a common pattern in multidimensional food-behavior scales, where short, content-specific factors trade internal consistency for breadth of coverage. By reporting McDonald’s omega alongside Cronbach’s alpha, by formally comparing competing factor structures, and by evaluating discriminant validity through both the Fornell-Larcker and HTMT criteria, the present study provides a more complete psychometric characterization than earlier bean-specific tools, which generally reported reliability alone. The convergence of these indices on a correlated four-factor structure, together with the cross-validation results, strengthens confidence in the novelty and robustness of the instrument relative to the existing literature.

Beyond its measurement properties, the instrument has clear public health and sustainability relevance. Beans are among the most nutrient-dense, affordable, and environmentally sustainable protein sources available, and increasing their consumption is widely promoted as a means of improving diet quality while reducing reliance on resource-intensive animal protein (Didinger & Thompson, 2022; Kaartinen et al., 2023; Sranacharoenpong et al., 2015). A validated, theory-based survey provides a practical way to operationalize these goals in young-adult populations. In applied terms, the tool can serve as a baseline assessment for nutrition and sustainability interventions, as a means of monitoring change in knowledge, attitudes, and behaviors over time, and as an outcome measure for campus-based programs such as nutrition education, dining-hall menu redesign, and food-environment initiatives. Because it captures distinct behavioral, attitudinal, and comparative-evaluation dimensions, it can help intervention designers target the specific determinants most in need of change rather than treating bean consumption as a single undifferentiated outcome.

Several directions for future research follow from these findings. First, external validation in more diverse populations—including students at different universities, community-college and non-student young adults, and culturally and ethnically varied groups—is needed before the tool can be applied broadly, given the predominantly White and female sample studied here. Second, longitudinal studies linking survey scores to objectively measured dietary intake (for example, food-purchase or consumption records) would establish predictive and criterion validity. Third, intervention studies using the instrument as a repeated outcome measure would test its responsiveness to change. Finally, the comparatively low AVE values and the two factor pairs that did not meet the Fornell-Larcker criterion indicate that targeted item revision and re-estimation of the model in independent samples should be a priority, ideally complemented by formal test-retest assessment of temporal stability, which the present cross-sectional design could not provide.

## Conclusions

Despite these limitations, this research produced a reliable and valid tool that can be used to assess college students’ knowledge, attitudes and behaviors regarding beans, helping to fill a gap in legume-specific dietary assessment. The instrument has practical value for research, intervention, and monitoring, offering a theory-based means of establishing baseline bean-related characteristics, tracking change in knowledge, attitudes, and behaviors over time, and evaluating the effectiveness of bean-promotion interventions in college settings. Its relevance extends beyond food insecurity to broader public health and sustainability goals, given the role of beans as a nutrient-dense, affordable, and environmentally sustainable protein source and the value of shifting young adults toward more plant-forward eating patterns. With further validation, the tool may also be applicable to other populations. Taken together, this instrument can support future studies and strategies aimed at improving diet quality and promoting sustainable dietary patterns among young adults.

## Supplemental Information

10.7717/peerj.21881/supp-1 Supplemental Information 1 Scree plot of eigenvalues with parallel analysis Eigenvalues from the correlation matrix of 24 items (*N* = 134). The horizontal reference line indicates eigenvalue = 1. Parallel analysis suggested five factors initially; iterative refinement yielded a four-factor solution.

10.7717/peerj.21881/supp-2 Supplemental Information 2 Network analysis of survey items Correlation-based network (EBICglasso) of the 14 retained items. Nodes colored by factor: Consumption (blue), Purchasing (green), Perceived Benefits (orange), Comparative Evaluations (pink). Edge thickness represents correlation strength. Strong within-factor clustering supports the four-factor structure.

10.7717/peerj.21881/supp-3 Supplemental Information 3 Discriminant validity: bean score by demographic subgroups Composite bean score distributions across demographic subgroups (gender, age, academic year, food security status) displayed as box plots (median, IQR, and outliers). Scores differed significantly by gender (*p* = .021) and food security status (*p* = .038, Kruskal–Wallis), but not by age group or academic year.

10.7717/peerj.21881/supp-4 Supplemental Information 4 Scatterplot: sHEI total score *vs.* composite bean score The nonsignificant correlation (*Pearsonr* = .027, *p* = .398) demonstrates that the bean consumption survey measures constructs distinct from general dietary quality as assessed by the sHEI.

10.7717/peerj.21881/supp-5 Supplemental Information 5 Original survey items with initial factor loadings and EFA disposition

10.7717/peerj.21881/supp-6 Supplemental Information 6 EFA variance explained and factor score adequacy for the final four-factor solution

10.7717/peerj.21881/supp-7 Supplemental Information 7 Cross-validation of the modified CFA model

10.7717/peerj.21881/supp-8 Supplemental Information 8 Item scoring scheme for bean consumption survey

10.7717/peerj.21881/supp-9 Supplemental Information 9 Discriminant validity: composite bean score by demographic subgroups

10.7717/peerj.21881/supp-10 Supplemental Information 10 Correlation with short healthy eating index (sHEI)

10.7717/peerj.21881/supp-11 Supplemental Information 11 Psychometric scale codeboo all variables from the survey instrument, organized into three psychometric domains: Knowledge Items (K1–K3) assessing factual understanding of bean nutrition and preparation; Attitude Items (A1–A10) measuring beliefs about health benefits, affordability, taste, cooking confidence, and environmental impact; and Behavior Items (B1–B16) capturing frequency and context of bean consumption and food preparation practices. Response codes follow a standardized 1–5 Likert scale ranging from negative positions (Never, Strongly Disagree) to positive positions (Always/Very Often, Strongly Agree), with items A9 reverse-scored. Items marked with (R) indicate reverse-coded questions requiring rescaling prior to analysis.

10.7717/peerj.21881/supp-12 Supplemental Information 12 Item-level descriptive statistics for the 14 retained items (CFA sample, *N* = 732)

10.7717/peerj.21881/supp-13 Supplemental Information 13 Confirmatory factor analysis sample (*n* = 732) 732 respondent observations from an independent sample used for confirmatory factor analysis, allowing cross-validation and generalizability testing of the factor structure derived from the exploratory phase. Raw response data (1–5 scale) for all 32 items are provided with missing values indicated by blanks.

10.7717/peerj.21881/supp-14 Supplemental Information 14 Factor correlations and effect size interpretations (modified CFA model)

10.7717/peerj.21881/supp-15 Supplemental Information 15 Exploratory factor analysis sample (*n* = 250): raw response data for consumer bean consumption knowledge, attitudes, and behavioral scales 250 respondent records used in the exploratory factor analysis phase to identify underlying latent factor structures. All 32 items are presented in raw response format (values 1–5 corresponding to codebook response categories), with missing values represented by blanks indicating incomplete responses or non-applicable items.

10.7717/peerj.21881/supp-16 Supplemental Information 16 Measurement invariance testing across bean consumption groups (high *vs.* low)

10.7717/peerj.21881/supp-17 Supplemental Information 17 Statistical power analysis for CFA

10.7717/peerj.21881/supp-18 Supplemental Information 18 Largest standardized residual correlations from the modified CFA model

## Additional Information and Declarations

### Data Availability

The following information was supplied regarding data availability: The data is available in the Supplemental Files.
