# Supplementary material for: Development and validation of a bean knowledge, attitudes, and behaviors survey in a college population
Source: PeerJ. 2026 Oct 26;14:e21881. doi: 10.7717/peerj.21881 (PMC13718447; doi:10.7717/peerj.21881)
Supplement: Supplementary file 1 [file peerj-14-21881-s001.pdf]

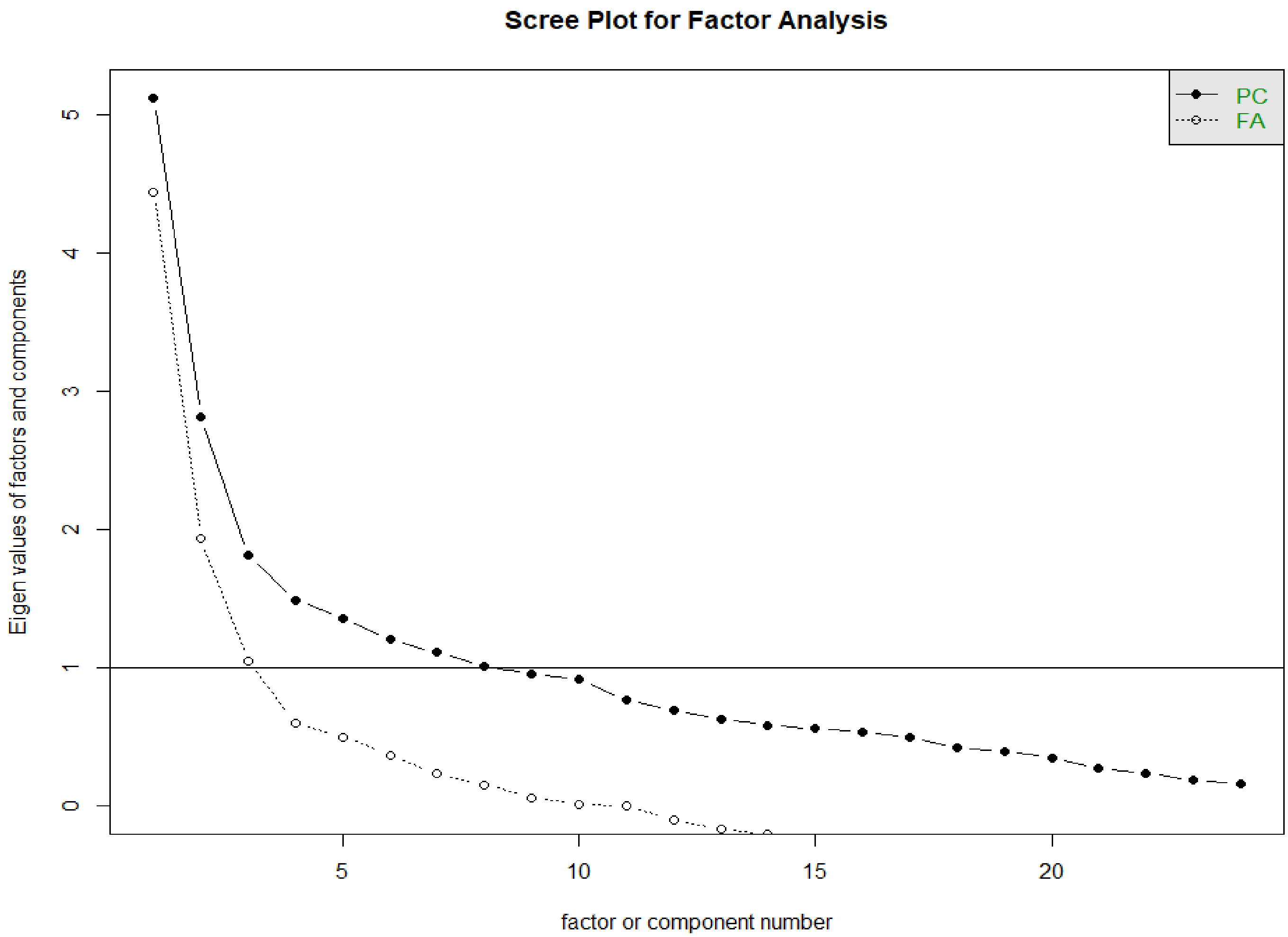

Figure S1. *Scree Plot of Eigenvalues with Parallel Analysis*

Note. Eigenvalues from the correlation matrix of 24 items (N = 134). The horizontal reference line indicates eigenvalue = 1. Parallel analysis suggested five factors initially; iterative refinement yielded a four-factor solution.
