# Supplementary material for: Development and validation of a bean knowledge, attitudes, and behaviors survey in a college population
Source: PeerJ. 2026 Oct 26;14:e21881. doi: 10.7717/peerj.21881 (PMC13718447; doi:10.7717/peerj.21881)
Supplement: Supplementary file 2 [file peerj-14-21881-s002.pdf]

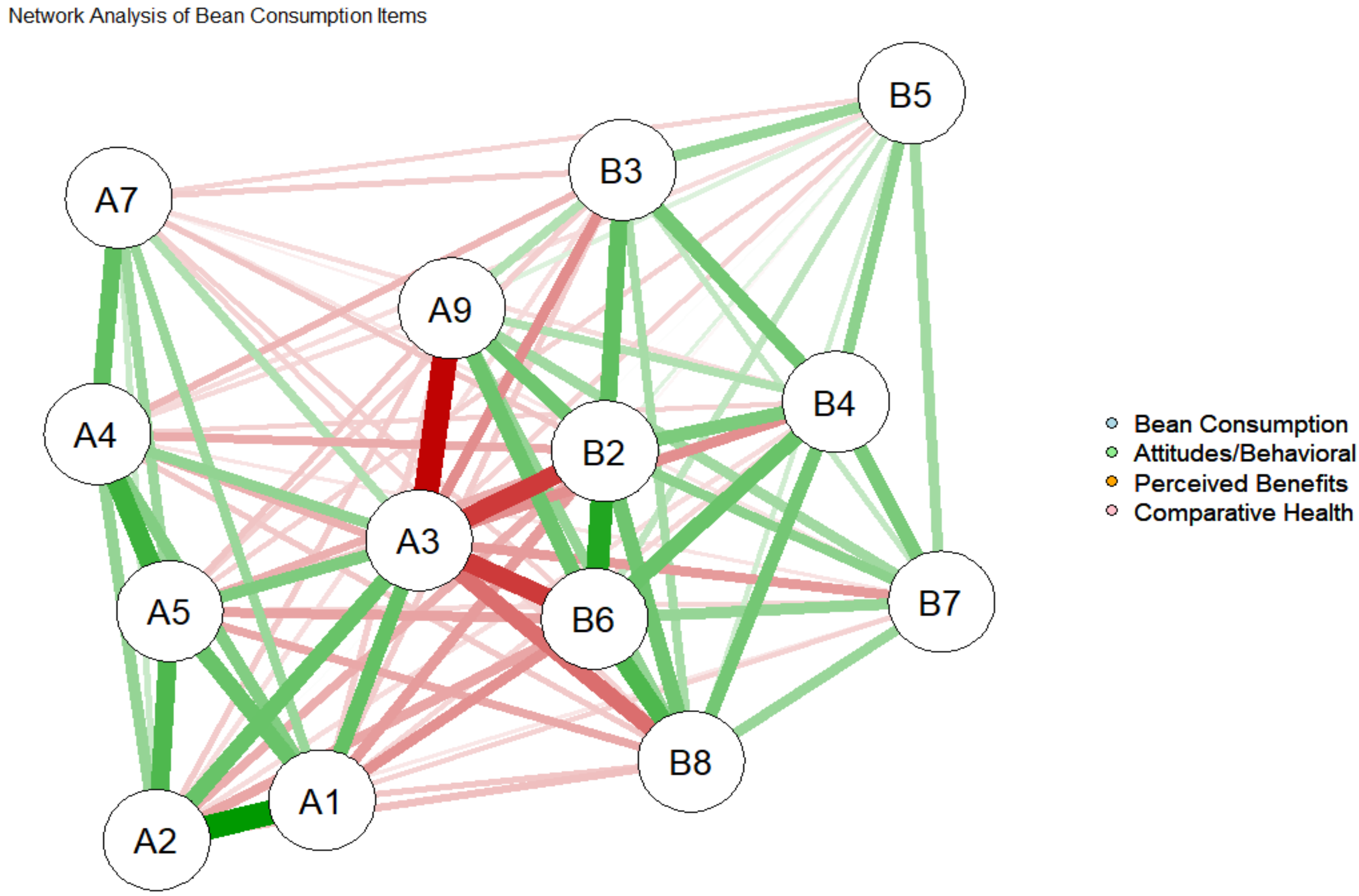

Figure S2. *Network Analysis of Bean Survey Items*

Note. Correlation-based network (EBICglasso) of the 14 retained items. Nodes colored by factor: Consumption (blue), Purchasing (green), Perceived Benefits (orange), Comparative Evaluations (pink). Edge thickness represents correlation strength. Strong within-factor clustering supports the four-factor structure.
