# Supplementary material for: Development and validation of a bean knowledge, attitudes, and behaviors survey in a college population
Source: PeerJ. 2026 Oct 26;14:e21881. doi: 10.7717/peerj.21881 (PMC13718447; doi:10.7717/peerj.21881)
Supplement: Supplementary file 3 [file peerj-14-21881-s003.pdf]

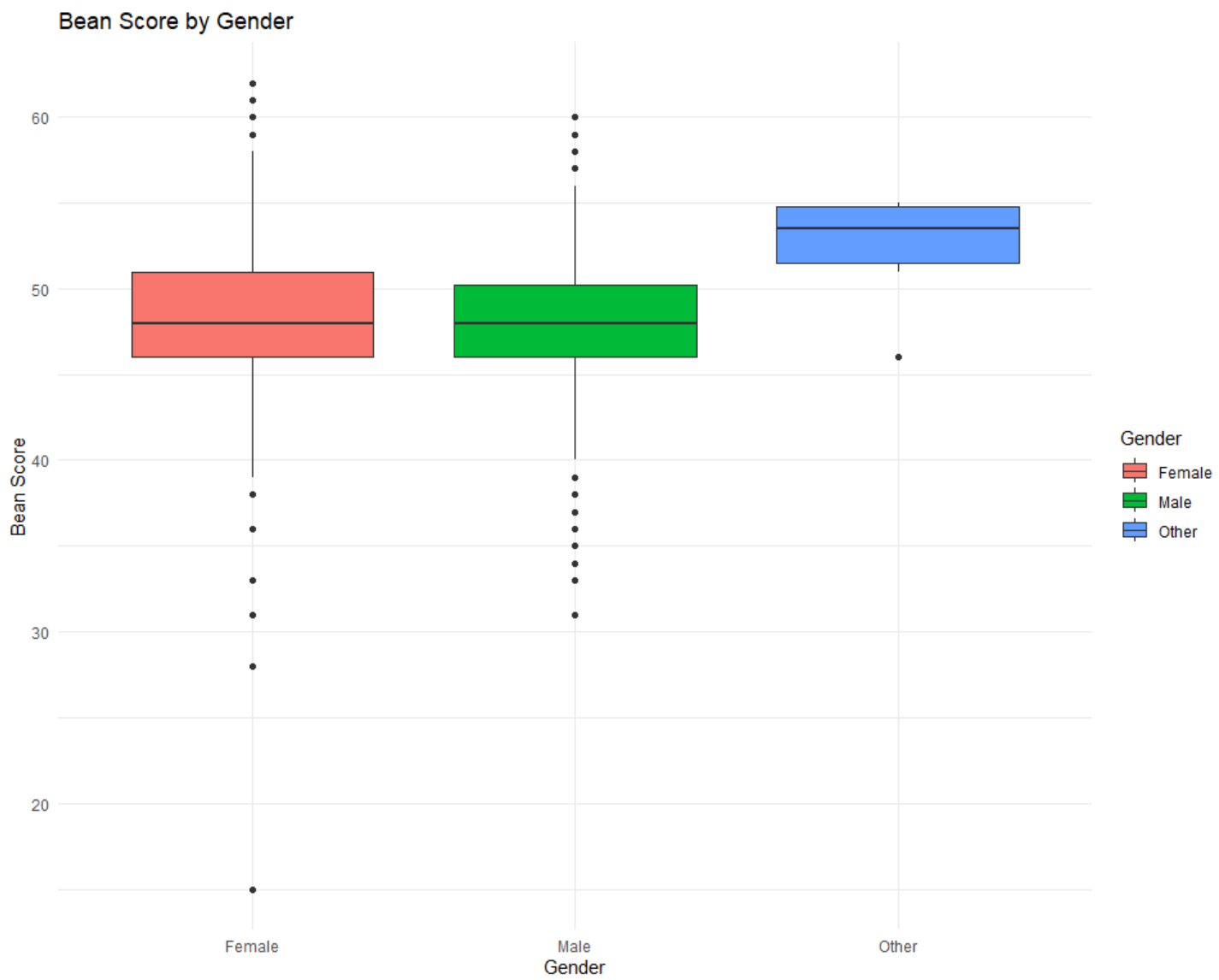

Panel A: Bean Score by Gender

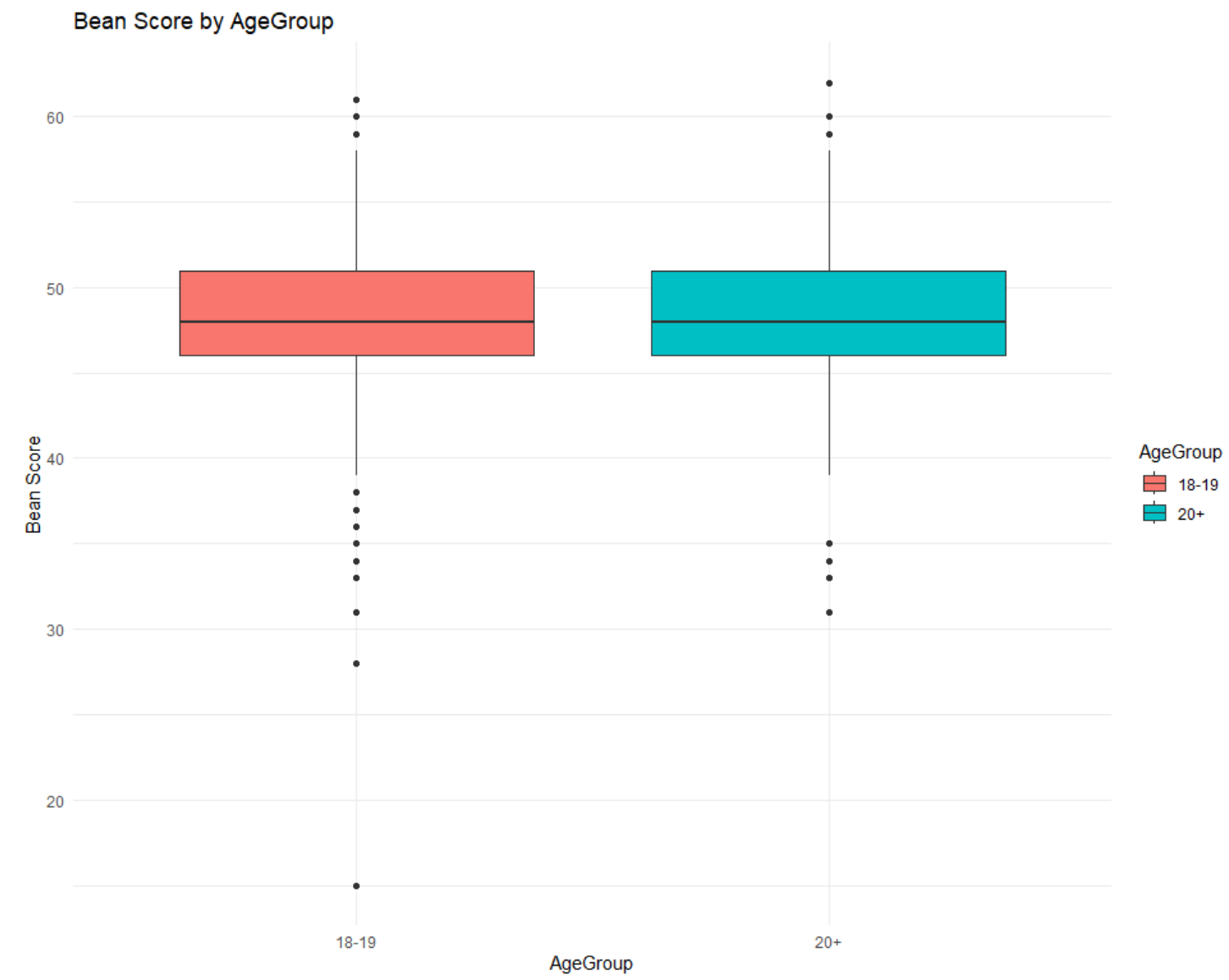

Panel B: Bean Score by Age Group

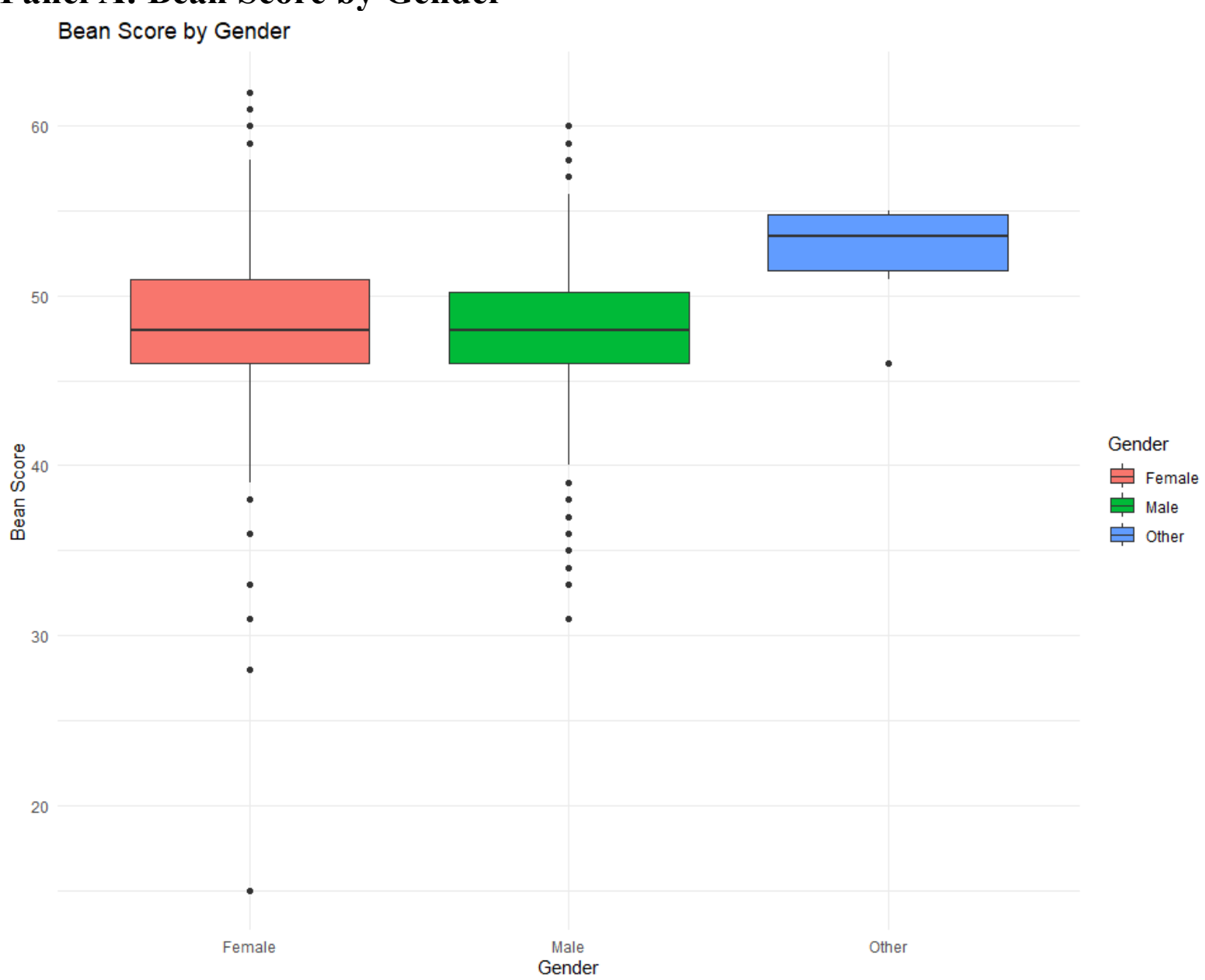

Panel C: Bean Score by Academic Year

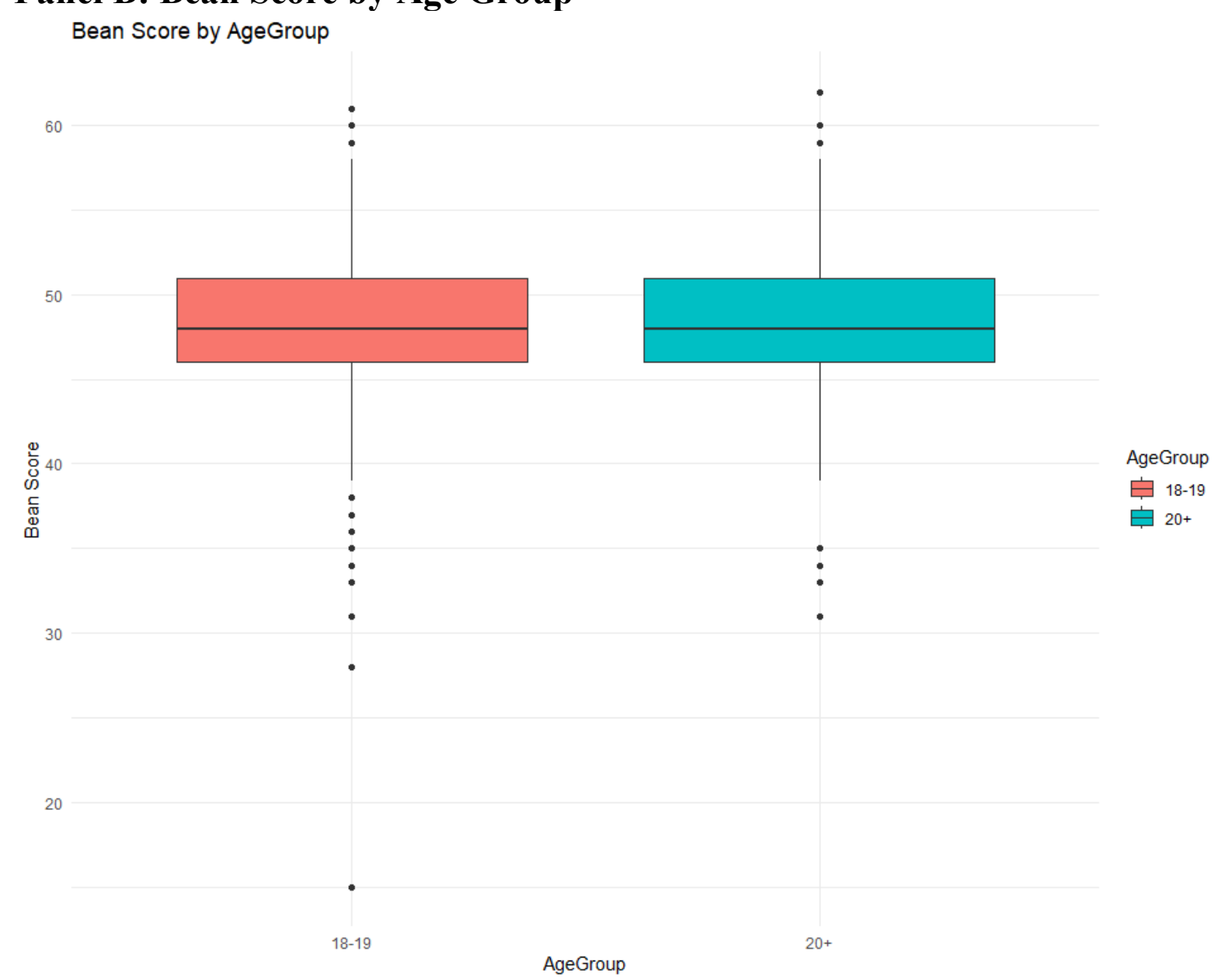

Panel D: Bean Score by Food Security Status

Figure S3. *Discriminant Validity: Bean Score by Demographic Subgroups*

Note. Box plots showing composite bean score distributions across demographic subgroups. Significant differences found for gender ($p = .021$) and food security status ($p = .038$, Kruskal-Wallis). See Supplementary Table S5 for detailed statistics.
