# Supplementary material for: Development and validation of a bean knowledge, attitudes, and behaviors survey in a college population
Source: PeerJ. 2026 Oct 26;14:e21881. doi: 10.7717/peerj.21881 (PMC13718447; doi:10.7717/peerj.21881)
Supplement: Supplementary file 4 [file peerj-14-21881-s004.pdf]

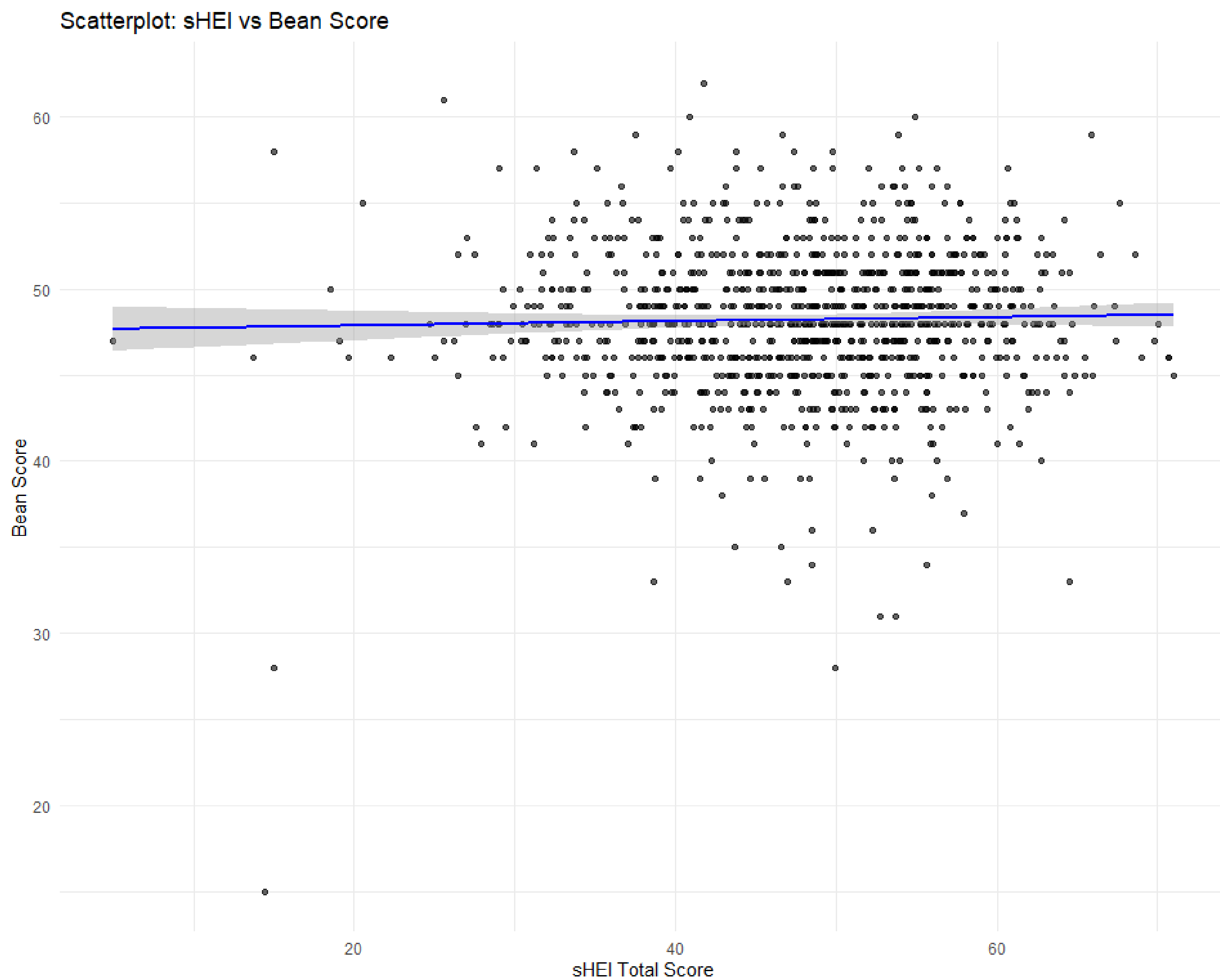

Figure S4. *Scatterplot: sHEI Total Score vs. Composite Bean Score*

Note. Pearson r = .027, p = .398. The negligible, nonsignificant correlation demonstrates that the bean consumption survey measures constructs distinct from general dietary quality as assessed by the sHEI.
