# Supplementary material for: Development and validation of a bean knowledge, attitudes, and behaviors survey in a college population
Source: PeerJ. 2026 Oct 26;14:e21881. doi: 10.7717/peerj.21881 (PMC13718447; doi:10.7717/peerj.21881)
Supplement: Supplementary file 5 [file peerj-14-21881-s005.docx]

**Table S1.** *Original Survey Items with Initial Factor Loadings and EFA Disposition*

| **Item** | **Construct** | **Item Text** | **ML3** | **ML2** | **ML5** | **ML1** | **ML4** | ***h*²** | **Disposition** | **Final Factor / Reason** |
| --- | --- | --- | --- | --- | --- | --- | --- | --- | --- | --- |
| ***Panel A: Retained Items (n = 14)*** |  |  |  |  |  |  |  |  |  |  |
| A9 | Attitudes | How much do you agree: I do not like the texture of beans (R) | .17 | −.03 | .41 | .60 | .23 | .61 | Retained | F1: Consumption |
| A3 | Attitudes | How much do you agree: I like the taste of beans | −.19 | .39 | −.15 | −.88 | .10 | 1.00 | Retained | F1: Consumption |
| B6 | Behavior | How often do you consume beans as a side dish? | .20 | −.19 | .59 | .37 | −.18 | .59 | Retained | F1: Consumption |
| B2 | Behavior | On average, how often do you eat beans/products made from beans? | .23 | −.11 | .58 | .32 | −.05 | .51 | Retained | F1: Consumption |
| B5 | Behavior | How often do you consume beans as a dessert? | .79 | .02 | −.09 | .05 | .04 | .63 | Retained | F2: Purchasing |
| B4 | Behavior | How often do you buy canned beans? | .63 | −.06 | .20 | .22 | −.07 | .50 | Retained | F2: Purchasing |
| B7 | Behavior | How often do you consume beans as a main dish? | .59 | −.05 | .15 | .08 | −.14 | .40 | Retained | F2: Purchasing |
| B8 | Behavior | How often do you use beans as an ingredient during cooking? | .58 | −.08 | .23 | .23 | −.03 | .45 | Retained | F2: Purchasing |
| B3 | Behavior | How often do you consume beans as a dip? | .52 | −.16 | .19 | .25 | .06 | .40 | Retained | F2: Purchasing |
| A2 | Attitudes | How much do you agree: Beans are affordable | −.06 | .88 | −.01 | −.08 | .10 | .80 | Retained | F3: Perc. Benefits |
| A1 | Attitudes | How much do you agree: Beans are good for my health | −.04 | .88 | −.01 | −.03 | .13 | .80 | Retained | F3: Perc. Benefits |
| A5 | Attitudes | How much do you agree: Buying beans saves me money | −.16 | .44 | −.02 | −.22 | .33 | .38 | Retained | F3: Perc. Benefits |
| A4 | Attitudes | How much do you agree: Beans are better for the environment than meat | −.05 | .15 | −.01 | −.08 | .86 | .78 | Retained | F4: Comp. Eval. |
| A7 | Attitudes | How much do you agree: Beans can be as healthy as meat | −.07 | .09 | .04 | .05 | .58 | .36 | Retained | F4: Comp. Eval. |
| ***Panel B: Removed Items (n = 10)*** |  |  |  |  |  |  |  |  |  |  |
| K1 | Knowledge | Bean types and identification | .23 | −.03 | .00 | −.10 | −.01 | .06 | Removed (Iter. 1) | Low loading (<.40) |
| K2 | Knowledge | Bean serving size knowledge | .07 | −.17 | .08 | −.04 | −.19 | .08 | Removed (Iter. 1) | Low loading (<.40) |
| K3 | Knowledge | Bean nutritional benefits knowledge | −.17 | .03 | −.09 | .04 | −.10 | .05 | Removed (Iter. 1) | Low loading (<.40) |
| B9 | Behavior | How often do you consume beans as a snack? | .38 | .01 | .13 | .17 | −.07 | .20 | Removed (Iter. 2) | Low communality (.20) |
| B14 | Behavior | How often do you prepare dried beans at home? | .25 | −.03 | .53 | .25 | .00 | .40 | Removed (Iter. 2) | Cross-loading (ML3/ML5) |
| B15 | Behavior | Do you know how to cook dried beans? | .12 | .23 | −.14 | −.15 | .21 | .15 | Removed (Iter. 2) | Low communality (.15) |
| A8 | Attitudes | How much do you agree: I know how to prepare beans in multiple ways | −.12 | .28 | −.07 | −.09 | .00 | .11 | Removed (Iter. 3) | Loading <.40 all factors |
| B1 | Behavior | How often do you eat beans in a typical week? | −.18 | −.35 | −.36 | −.17 | −.09 | .32 | Removed (Iter. 4) | Cross-load; no clean factor |
| A6 | Attitudes | How much do you agree: Beans take too long to cook | .10 | .01 | .09 | .19 | .23 | .11 | Removed (Iter. 4) | Loading <.40 all factors |
| A10 | Attitudes | How much do you agree: I am confident in my ability to cook beans | .04 | −.02 | .59 | −.08 | .06 | .36 | Removed (Iter. 5) | Near-zero h² in final |

*Note.* Loadings from initial 5-factor EFA (varimax rotation, ML extraction) before iterative refinement. (R) = reverse-coded. Iter. = iteration of removal. Primary loading threshold = .40; cross-loading threshold = .32. Iteration 1: K1–K3 (low loadings). Iteration 2: B9, B14, B15 (low communalities/cross-loading). Iteration 3: A8 (no factor ≥.40). Iteration 4: B1, A6 (no clean assignment; model reduced to 4 factors). Iteration 5: A10 (near-zero communality).
