# Supplementary material for: Development and validation of a bean knowledge, attitudes, and behaviors survey in a college population
Source: PeerJ. 2026 Oct 26;14:e21881. doi: 10.7717/peerj.21881 (PMC13718447; doi:10.7717/peerj.21881)
Supplement: Supplementary file 6 [file peerj-14-21881-s006.docx]

**Table S2**

*EFA Variance Explained and Factor Score Adequacy for the Final Four-Factor Solution*

| **Statistic** | **Consumption (ML3)** | **Purchasing (ML4)** | **Perc. Benefits (ML2)** | **Comp. Eval. (ML1)** |
| --- | --- | --- | --- | --- |
| SS Loadings | 2.30 | 2.13 | 1.97 | 1.46 |
| Proportion of Variance | 0.16 | 0.15 | 0.14 | 0.10 |
| Cumulative Variance | 0.16 | 0.32 | 0.46 | 0.56 |
| Proportion Explained | 0.29 | 0.27 | 0.25 | 0.19 |
| Cumulative Proportion Explained | 0.29 | 0.56 | 0.81 | 1.00 |
| Corr. of Scores with Factors | 0.90 | 0.87 | 0.94 | 1.00 |
| Multiple R² of Scores | 0.81 | 0.76 | 0.88 | 0.99 |
| Min. Corr. of Factor Scores | 0.62 | 0.52 | 0.77 | 0.98 |

*Note.* Final four-factor solution after iterative removal of 10 items. Varimax rotation, ML extraction. Total variance explained = 56.1%. RMSR = .04; df-corrected RMSR = .06.
