# Supplementary material for: Development and validation of a bean knowledge, attitudes, and behaviors survey in a college population
Source: PeerJ. 2026 Oct 26;14:e21881. doi: 10.7717/peerj.21881 (PMC13718447; doi:10.7717/peerj.21881)
Supplement: Supplementary file 7 [file peerj-14-21881-s007.docx]

**Table S3.**

*Cross-Validation of the Modified CFA Model*

| **Sample** | **N** | **CFI** | **TLI** | **RMSEA** | **SRMR** | **ΔCFI** |
| --- | --- | --- | --- | --- | --- | --- |
| Training (70%) | 512 | .909 | .881 | .073 | .051 | — |
| Validation (30%) | 220 | .908 | .878 | .076 | .062 | .001 |
| Full Sample | 732 | .914 | .886 | .071 | .047 | — |

*Note.* 70/30 random split (seed = 123). ΔCFI between training and validation = .001, well below the .01 threshold for invariance concerns (Cheung & Rensvold, 2002). Consistent fit indices across subsamples support model generalizability.
