# Supplementary material for: Development and validation of a bean knowledge, attitudes, and behaviors survey in a college population
Source: PeerJ. 2026 Oct 26;14:e21881. doi: 10.7717/peerj.21881 (PMC13718447; doi:10.7717/peerj.21881)
Supplement: Supplementary file 8 [file peerj-14-21881-s008.docx]

**Table S4.** *Item Scoring Scheme for Bean Consumption Survey*

| **Item** | **Type** | **Question** | **Response Scale** | **Scoring** |
| --- | --- | --- | --- | --- |
| A9 | Agreement (R) | I do not like the texture of beans | SA=1 to SD=5 | Reverse: 5,4,3,2,1 |
| A3 | Agreement | I like the taste of beans | SD=1 to SA=5 | Direct: 1,2,3,4,5 |
| B2 | Frequency | How often do you eat beans/bean products? | Often=1 to Never=5, Opt-out=6 | Direct: 1–5; 6→0 |
| B3 | Frequency | How often do you consume beans as a dip? | Most Days=1 to Never=5, Opt-out=6 | Direct: 1–5; 6→0 |
| B4 | Frequency | How often do you buy canned beans? | Most Days=1 to Never=5, Opt-out=6 | Direct: 1–5; 6→0 |
| B5 | Frequency | How often do you consume beans as a dessert? | Most Days=1 to Never=5, Opt-out=6 | Direct: 1–5; 6→0 |
| B6 | Frequency | How often do you consume beans as a side dish? | Most Days=1 to Never=5, Opt-out=6 | Direct: 1–5; 6→0 |
| B7 | Frequency | How often do you consume beans as a main dish? | Most Days=1 to Never=5, Opt-out=6 | Direct: 1–5; 6→0 |
| B8 | Frequency | How often do you use beans as an ingredient? | Most Days=1 to Never=5, Opt-out=6 | Direct: 1–5; 6→0 |
| A1 | Agreement | Beans are good for my health | SD=1 to SA=5 | Direct: 1,2,3,4,5 |
| A2 | Agreement | Beans are affordable | SD=1 to SA=5 | Direct: 1,2,3,4,5 |
| A5 | Agreement | Buying beans saves me money | SD=1 to SA=5 | Direct: 1,2,3,4,5 |
| A4 | Agreement | Beans are better for the environment than meat | SD=1 to SA=5 | Direct: 1,2,3,4,5 |
| A7 | Agreement | Beans can be as healthy as meat | SD=1 to SA=5 | Direct: 1,2,3,4,5 |

*Note.* (R) = reverse coded. SA = Strongly Agree; SD = Strongly Disagree. Scores of 0 (opt-out) are excluded from factor score calculations. Factor scores = mean of valid non-zero responses within each factor. Items grouped by factor: Consumption (A9, A3, B6, B2), Purchasing (B5, B4, B7, B8, B3), Perceived Benefits (A2, A1, A5), Comparative Evaluations (A4, A7).
