# Supplementary material for: Development and validation of a bean knowledge, attitudes, and behaviors survey in a college population
Source: PeerJ. 2026 Oct 26;14:e21881. doi: 10.7717/peerj.21881 (PMC13718447; doi:10.7717/peerj.21881)
Supplement: Supplementary file 9 [file peerj-14-21881-s009.docx]

**Table S5.** *Discriminant Validity: Composite Bean Score by Demographic Subgroups*

| **Variable** | **Category** | **n** | **M (SD)** | **F** | **p** | **χ² KW** | **p KW** | **η²** |
| --- | --- | --- | --- | --- | --- | --- | --- | --- |
| Gender | Female | 644 | 39.2 (4.3) | 3.87 | .021* | 8.33 | .016* | .008 |
|  | Male | 332 | 38.1 (4.5) |  |  |  |  |  |
|  | Other | 6 | 37.5 (5.1) |  |  |  |  |  |
| Age Group | 18–19 | 683 | 38.7 (4.4) | 0.64 | .425 | 1.74 | .188 | .001 |
|  | 20+ | 299 | 38.5 (4.3) |  |  |  |  |  |
| Year | Freshman | 456 | 38.8 (4.4) | 0.54 | .657 | 1.06 | .788 | .002 |
|  | Sophomore | 350 | 38.5 (4.4) |  |  |  |  |  |
|  | Junior | 118 | 38.5 (4.2) |  |  |  |  |  |
|  | Senior | 58 | 39.0 (4.6) |  |  |  |  |  |
| Food Security | High/Marginal | 431 | 38.8 (4.3) | 2.65 | .071† | 6.52 | .038* | .005 |
|  | Low | 351 | 38.9 (4.3) |  |  |  |  |  |
|  | Very Low | 200 | 38.1 (4.6) |  |  |  |  |  |
