# Supplementary material for: Development and validation of a bean knowledge, attitudes, and behaviors survey in a college population
Source: PeerJ. 2026 Oct 26;14:e21881. doi: 10.7717/peerj.21881 (PMC13718447; doi:10.7717/peerj.21881)
Supplement: Supplementary file 10 [file peerj-14-21881-s010.docx]

**Table S5b**

*Correlation with Short Healthy Eating Index (sHEI)*

| **Measure** | ***r*** | ***t*** | ***df*** | ***p*** |
| --- | --- | --- | --- | --- |
| sHEI Total Score | .027 | 0.845 | 980 | .398 |

*Note.* * p < .05. † p < .10. KW = Kruskal-Wallis. η² = eta-squared from ANOVA. Pearson correlation between composite bean score and sHEI total score was nonsignificant (r = .027, p = .398), supporting discriminant validity.
