# Supplementary material for: Development and validation of a bean knowledge, attitudes, and behaviors survey in a college population
Source: PeerJ. 2026 Oct 26;14:e21881. doi: 10.7717/peerj.21881 (PMC13718447; doi:10.7717/peerj.21881)
Supplement: Supplementary file 11 [file peerj-14-21881-s012.docx]

**Table S6.**

*Item-Level Descriptive Statistics for the 14 Retained Items (CFA Sample, N = 732)*

| **Item** | **M** | **SD** | **Range** | **Skewness** | **Kurtosis** | **Factor** |
| --- | --- | --- | --- | --- | --- | --- |
| B2 | 2.10 | 1.15 | 1–6 | 1.18 | 0.82 | Consumption |
| B3 | 3.79 | 0.90 | 1–6 | −0.28 | −0.18 | Purchasing |
| B4 | 4.31 | 0.94 | 1–6 | −1.12 | 0.36 | Purchasing |
| B5 | 4.90 | 0.42 | 1–6 | −4.68 | 26.86 | Purchasing |
| B6 | 3.40 | 0.94 | 1–6 | 0.08 | −0.31 | Consumption |
| B7 | 4.48 | 0.85 | 1–6 | −1.51 | 1.65 | Purchasing |
| B8 | 4.09 | 0.87 | 1–6 | −0.45 | −0.49 | Purchasing |
| A1 | 4.08 | 0.65 | 1–5 | −1.00 | 4.03 | Perc. Benefits |
| A2 | 4.15 | 0.67 | 1–5 | −1.00 | 3.38 | Perc. Benefits |
| A3 | 3.48 | 1.18 | 1–5 | −0.69 | −0.40 | Consumption |
| A4 | 3.30 | 0.93 | 1–5 | −0.15 | 0.11 | Comp. Eval. |
| A5 | 3.44 | 0.85 | 1–5 | −0.04 | 0.09 | Perc. Benefits |
| A7 | 3.04 | 0.91 | 1–5 | −0.09 | −0.26 | Comp. Eval. |
| A9 | 2.78 | 1.27 | 1–5 | 0.31 | −1.04 | Consumption |

*Note.* B5 (beans as dessert) showed extreme kurtosis (26.86) due to ceiling effects; most respondents reported never consuming beans as a dessert. Mardia’s test indicated significant multivariate non-normality (skewness b1p = 39.33, p < .001; kurtosis b2p = 286.82, p < .001), supporting the use of MLR estimation in CFA.
