# Supplementary material for: Development and validation of a bean knowledge, attitudes, and behaviors survey in a college population
Source: PeerJ. 2026 Oct 26;14:e21881. doi: 10.7717/peerj.21881 (PMC13718447; doi:10.7717/peerj.21881)
Supplement: Supplementary file 12 [file peerj-14-21881-s014.docx]

**Table S7**

*Factor Correlations and Effect Size Interpretations (Modified CFA Model)*

| **Factor** | **1** | **2** | **3** | **4** |
| --- | --- | --- | --- | --- |
| **1. Consumption** | 1.00 |  |  |  |
| **2. Purchasing** | .816*** | 1.00 |  |  |
| **3. Perceived Benefits** | −.360*** | −.532*** | 1.00 |  |
| **4. Comparative Evaluations** | −.295*** | −.374*** | .734*** | 1.00 |

*Note.* *** p < .001. Effect sizes: |r| < .30 = small; .30 ≤ |r| < .50 = medium; |r| ≥ .50 = large (Cohen, 1988). Consumption–Purchasing = large; Purchasing–Perc. Benefits = large; Perc. Benefits–Comp. Eval. = large; Consumption–Perc. Benefits = medium; Consumption–Comp. Eval. = small; Purchasing–Comp. Eval. = medium.
