# Supplementary material for: Development and validation of a bean knowledge, attitudes, and behaviors survey in a college population
Source: PeerJ. 2026 Oct 26;14:e21881. doi: 10.7717/peerj.21881 (PMC13718447; doi:10.7717/peerj.21881)
Supplement: Supplementary file 13 [file peerj-14-21881-s016.docx]

**Table S8**

*Measurement Invariance Testing Across Bean Consumption Groups (High vs. Low)*

| **Model** | **χ²** | **df** | **CFI** | **RMSEA** | **Δχ² (df)** | **p** |
| --- | --- | --- | --- | --- | --- | --- |
| Configural | 366.33 | 138 | .886 | .067 | — | — |
| Metric | 468.80 | 148 | .840 | .077 | 71.60 (10) | <.001*** |
| Scalar | 532.62 | 158 | .814 | .080 | 34.26 (10) | <.001*** |

*Note.* Groups formed by median split on total bean consumption (B items). *** p < .001. ΔCFI from configural to metric = .046; from metric to scalar = .026. Both exceed the .01 threshold (Cheung & Rensvold, 2002), indicating non-invariance of factor loadings and intercepts across high and low consumption groups. Results suggest the factor structure may operate differently across consumption levels, warranting caution when comparing group means.
