# Supplementary material for: Development and validation of a bean knowledge, attitudes, and behaviors survey in a college population
Source: PeerJ. 2026 Oct 26;14:e21881. doi: 10.7717/peerj.21881 (PMC13718447; doi:10.7717/peerj.21881)
Supplement: Supplementary file 14 [file peerj-14-21881-s017.docx]

**Table S9**

*Statistical Power Analysis for CFA*

| **Parameter** | **Value** |
| --- | --- |
| Test | RMSEA close-fit test (H₀: RMSEA ≤ .05 vs H₁: RMSEA ≥ .08) |
| Model degrees of freedom | 69 |
| Sample size (N) | 732 |
| Significance level (α) | .05 |
| Achieved power (1 − β) | 1.000 |
| Minimum N for 80% power | 79 |

*Note.* The CFA sample (N = 732) far exceeds the minimum sample size of 79 needed for 80% power to detect RMSEA departures from close fit. Statistical power was effectively 1.00 for the modified model.
