# Supplementary material for: Development and validation of a bean knowledge, attitudes, and behaviors survey in a college population
Source: PeerJ. 2026 Oct 26;14:e21881. doi: 10.7717/peerj.21881 (PMC13718447; doi:10.7717/peerj.21881)
Supplement: Supplementary file 15 [file peerj-14-21881-s018.docx]

**Table S10**

*Largest Standardized Residual Correlations from the Modified CFA Model*

| **Rank** | **Item 1** | **Item 2** | **\|Residual r\|** | **Within Factor?** |
| --- | --- | --- | --- | --- |
| 1 | A3 (Taste) | A1 (Health) | .194 | No (Cons./PB) |
| 2 | A3 (Taste) | A2 (Affordable) | .180 | No (Cons./PB) |
| 3 | B5 (Dessert) | B2 (Frequency) | .117 | No (Purch./Cons.) |
| 4 | B3 (Dip) | B2 (Frequency) | .098 | No (Purch./Cons.) |
| 5 | B8 (Ingredient) | B6 (Side dish) | .095 | No (Purch./Cons.) |
| 6 | B5 (Dessert) | B3 (Dip) | .089 | Yes (Purchasing) |
| 7 | B2 (Frequency) | A5 (Saves money) | .087 | No (Cons./PB) |
| 8 | B7 (Main dish) | B3 (Dip) | .087 | Yes (Purchasing) |
| 9 | B5 (Dessert) | B7 (Main dish) | .082 | Yes (Purchasing) |
| 10 | B5 (Dessert) | A2 (Affordable) | .080 | No (Purch./PB) |

*Note.* Only 3 of 91 unique residual correlations exceeded |.10| (3.3%), indicating adequate local fit. The two largest residuals (A3–A1 and A3–A2) involve cross-factor pairs between Consumption and Perceived Benefits, consistent with the moderate factor correlation (r = −.360).
